# Survival and complications after neoadjuvant chemoradiotherapy versus neoadjuvant chemotherapy for locally advanced gastric cancer: a systematic review and meta-analysis

**DOI:** 10.3389/fonc.2023.1177557

**Published:** 2023-05-09

**Authors:** Youqi Zhu, Jiuzhou Chen, Xueqing Sun, Yufei Lou, Miao Fang, Fengjuan Zhou, Lei Zhang, Yong Xin

**Affiliations:** ^1^ Department of Radiation, the Affiliated Hospital of Xuzhou Medical University, Xuzhou, Jiangsu, China; ^2^ Department of Cancer Institute, Xuzhou Medical University, Xuzhou, Jiangsu, China; ^3^ Department of Radiation, the Second Affiliated Hospital of Xuzhou Medical University, Xuzhou, Jiangsu, China; ^4^ Department of Oncology, Suining County People’s Hospital, Xuzhou, Jiangsu, China

**Keywords:** locally advanced gastric cancer, neoadjuvant chemoradiotherapy, systematic review, meta-analysis, gastric cancer

## Abstract

**Background:**

There is increasing evidence that neoadjuvant chemoradiotherapy is superior to neoadjuvant chemotherapy for patients with locally advanced gastric cancer. However, a number of studies have come to the opposite conclusion. Therefore, our meta-analysis is to evaluate the efficacy and safety of neoadjuvant chemoradiotherapy versus neoadjuvant chemotherapy in the treatment of locally advanced gastric cancer.

**Methods:**

We searched Wanfang Database, China National Knowledge Network database, VIP database, China Biomedical Literature Database, PubMed, Embase and Cochrane Library. The searched terms included’Stomach Neoplasms’, ‘Neoadjuvant Therapy’ and ‘Chemoradiotherapy’. The retrieval time was from the establishment of the corresponding database to September 2022, and our meta-analysis was performed using RevMan (version 5.3) and Stata (version 17) software.

**Results:**

A total of 17 literatures were included, which involved 7 randomized controlled trials and 10 retrospective studies, with a total of 6831 patients. The results of meta-analysis showed that compared with NACT group, the complete response rate(RR=1.95, 95%CI 1.39-2.73, p=0.0001), the partial response rate(RR=1.44, 95%CI 1.22-1.71, p=0.0001), the objective response rate(RR=1.37, 95%CI 1.27-1.54, p=0.00001), the pathologic complete response rate(RR=3.39, 95%CI 2.17-5.30, p=0.00001), the R0 resection rate(RR=1.18, 95%CI 1.09-1.29, p=0.0001) and 3-year overall survival rate(HR=0.89, 95%CI 0.82-0.96, p=0.002) of neoadjuvant chemoradiotherapy group were significantly improved. The results of subgroup analyses of gastric cancer subgroup and gastroesophageal junction cancer subgroup were consistent with the overall results. Meanwhile, the stable disease(RR=0.59, 95%CI:0.44-0.81, P=0.0010) of neoadjuvant chemoradiotherapy group was lower than that of neoadjuvant chemotherapy group, and there were no statistical significance in the progressive disease rate(RR=0.57, 95%CI:0.31-1.03, P=0.06), five-year overall survival rate(HR=1.03, 95%CI:0.99-1.07, P=0.839), postoperative complications and adverse reactions between the neoadjuvant chemoradiotherapy group and neoadjuvant chemotherapy group.

**Conclusion:**

Compared with neoadjuvant chemotherapy, neoadjuvant chemoradiotherapy might bring more survival benefits without significantly increasing adverse reactions. neoadjuvant chemoradiotherapy may be a recommended treatment for patients with locally advanced gastric cancer.

**Systematic Review Registration:**

https://inplasy.com/inplasy-2022-12-0068/, identifier INPLASY202212068.

## Introduction

Gastric cancer(GC) is the fifth most common type of cancer and the third leading cause of cancer-related death globally, with over 1,000,000 new cases and an estimated 783,000 deaths in 2020 (1). Worldwide, GC is the fourth most common malignant disease in males (fifth in females) and also the third leading cause of cancer death in men (fifth in women) (2).

For patients with locally advanced gastric cancer (LAGC), complete surgical resection is the only promising technique for curing the disease. In addition, the implementation of multi-mode therapy could also improve the survival chance of patients (3). Studies by Cats A and Hizal M et al. have confirmed that neoadjuvant therapy combined with surgical resection improved overall survival (OS) (4, 5). The National Comprehensive Cancer Network (NCCN) recommended neoadjuvant therapy for LAGC, neoadjuvant chemotherapy (NACT) and neoadjuvant chemoradiotherapy (NACRT) were both standard treatments (6, 7). Some studies have shown that NACRT could bring relatively high R0 resection and pathologic complete response (pCR) rate (3) and other researches also indicated that NACRT contributed to higher survival rate without significant increase in toxicity (8–10). On the contrary, some clinical trials pointed out that compared with NACT, NACRT failed to benefit the OS for LAGC patients (11–15). Therefore, whether NACRT could provide survival benefits remains controversial.

In recent years, with the development of radiotherapy technology, NACRT has become more and more popular. The addition of preoperative radiotherapy could enhance the killing of tumor cells in the primary tumor and metastatic tumor cells in the regional lymph nodes, thus reducing the local recurrence rate (16). Until now, preoperative treatment of LAGC is still a difficult problem for clinicians. This meta-analysis aims to systematically evaluate the efficacy and safety of NACRT versus NACT in the treatment of LAGC patients and hope to help clinical workers to choose the best regimens.

## Materials and methods

This systematic review and meta-analysis was based on a preplanned protocol constructed according to the standard Preferred Reporting Items for Systematic Reviews and MetaAnalysis (PRISMA) and was prospectively registered on inplasy.com (INPLASY protocol 2022120068. doi: 10.37766/inplasy2022.12.0068).

### Search strategy and study selection

To make our search more comprehensive, we searched Chinese and English databases. The Chinese databases included Wanfang Database, China National Knowledge Network Database, VIP Database and China Biomedical Literature Database. English databases include PubMed, Embase and Cochrane Library. At the same time, we searched relevant trials as of September 2022 in the international trial Registry platform and the Chinese Clinical Registry. We also reviewed the reference lists of included publications and of relevant review articles retrieved from the electronic searches to identify other potentially relevant studies that could have been missed. In PubMed and Embase, the search strategy we implemented was a combination of Medical Subject Headings (MESH) and various free text words for literature retrieval. Subject Headings used for the searching in PubMed were ‘**Stomach Neoplasms’**, ‘Neoadjuvant chemoradiotherapy’ and ‘Neoadjuvant chemotherapy’. A similar search strategy was performed in Embase but transformed according to the database’s thematic thesaurus. We used a combination of subject terms with keywords in the Cochrane Library and the remaining other databases were all searched using keywords.

### Inclusion and exclusion criteria

#### Inclusion criteria

(i) Type of study: Fully published randomized controlled trial(RCT) experiment or retrospective study. (ii) Subjects: Patients with surgically resectable LAGC with definite pathological diagnosis. (iii) Intervention measures: The experimental group was treated with NACRT and the control group was treated with NACT. (iv) Outcome indicators: The data of complete response (CR), partial response (PR), progressive disease (PD), stable disease (SD), objective response rate (ORR), pCR rate, R0 resection rate, incidence of postoperative adverse reactions and OS that were reported.

#### Exclusion criteria

(i) Studies that do not have access to full-text or republished studies. (ii) Reviews, systematic reviews, animal experiments, conference abstracts, case reports, one-arm studies. (iii) Studies in which outcome indicators were incomplete or unavailable. (iv) Breach of any of the above inclusion criteria.

### Quality assessment and risk of bias

Randomized controlled trials and retrospective studies were included in our meta-analysis. We evaluated the quality of the literatures using Cochrane Collaboration’s tool and the Newcastle-Ottawa scale (NOS) respectively. The CochraneCollaboration’s tool was scored on selection bias (randomized methods and assignment concealment), implementation bias (blinded investigators and subjects), implementation bias (blinded findings evaluation), follow-up bias (outcome data integrity), reporting bias (selective reporting of study results), and other biases. The NOS mainly included the selection (0- 4 stars), comparability (0- 2 stars), and outcome (0- 3 stars). If studies’ scores ≥ 6 stars, it would be regarded as high quality and enrolled in our meta-analysis.

### Data extraction

Two evaluators carefully read each document according to the inclusion and exclusion criteria and independently extract data from it. All the extracted data are checked repeatedly to ensure accuracy. If the data is disputed, a third evaluator will evaluate it. The main data contents extracted include: (i) general information: author, publication date and title; (ii) Intervention measures: chemotherapy and radiation dose; (iii) Outcome indexes: CR, PR, SD, PD, pCR rate, ORR, R0 resection rate, the incidence of postoperative complications, adverse reactions and OS.

### Statistical analysis

We used the RevMan software(version 5.3) and the Stata software (version 17) to conduct meta-analysis of the data obtained. The survival statistical analysis method of OS was inverted variance method, and the hazard ratio(HR) was used as the effect index. CR, PR, SD, PD, pCR rate, ORR, R0 resection rate, incidence of postoperative complications and incidence of adverse reactions were all dichotomous variables, and the risk ratio(RR) was used as the effect index. For the confidence interval (CI) of each effect index, 95%CI was used in this study, p<0.05 was considered statistically significant. The results of inter-study heterogeneity test were expressed by I^2^ value. If I^2^ ≤ 50% and p≥0.1, the heterogeneity between the studies was low, and the fixed-effect model was used for analysis. Otherwise, it indicates that there is obvious heterogeneity among the studies, therefore the random effects model is used for analysis. The funnel plot, egger test and begg test were used to evaluate the publication bias of the included studies.

## Results

### Characteristics of studies

**Figure 1:**
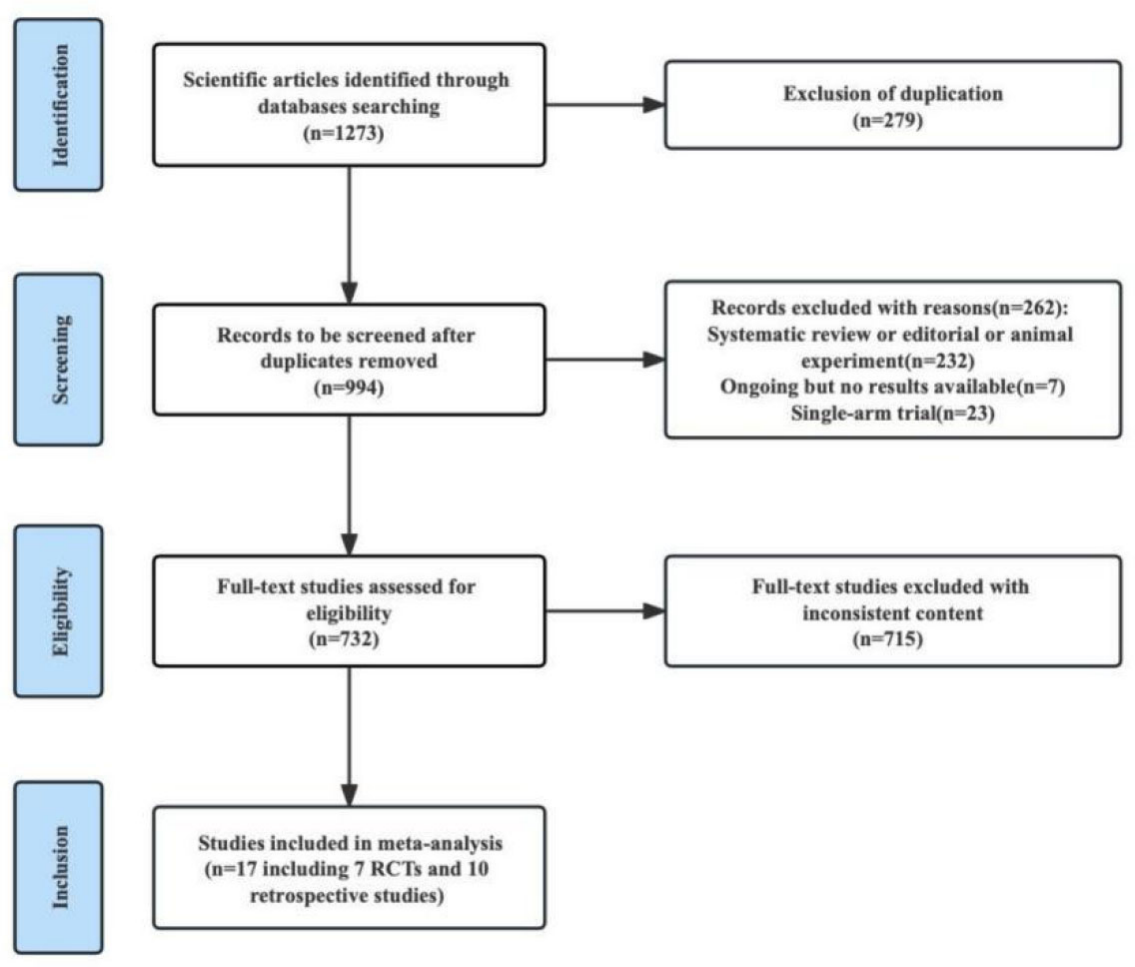
Flow chart of the studies selection process.

We identified 1273 studies through a search. 27 studies were obtained through preliminary screening. After reading the full text, 18 studies were finally included in our meta-analysis, including 7 RCTs and 11 retrospective studies. A flow chart of the literature screening is shown in **Figure 1**. There were a total of 7075 patients, including 4285 patients in the experimental group and 2790 patients in the control group. The detailed characteristics of each research were summed up in **Table 1**.

**Table 1:** Characteristics of included studies.

Study	Study design	Sample size (NACRT/NACT)	Gender (male/female)/			Age/years			Interventions		NOS score
			NACRT	NACT		NACRT		NACT	NACRT	NACT	
Zhang XT 2016 (17)	RCT	126(64/62)	78/48			55	57		S-1+ docetaxel + 45 Gy	S-1 + docetaxel	–
Cao MF 2019 (18)	RCT	59(29/30)	40/19			60.6 ± 7.1			TC(paclitaxel + carboplatin)+ 40 Gy	TC(paclitaxel + carboplatin)	–
Jiang Y 2019 (19)	RCT	84(42/42)	24/18		25/17	53.14 ± 8.72	53.14 ± 8.72		46.8–50.4 Gy concurrently with capecitabine	Oxaliplatin + capecitabine	–
He ZR 2017 (20)	RCT	50(25/25)	14/11		13/12	46.6 ± 4.5	47.7 ± 4.6		mFOLFOX-4(5-flfluorouracil + folinic acid + oxaliplatin) or capecitabine + 45 Gy	mFOLFOX-4(5- flfluorouracil + folinic acid + oxaliplatin) or capecitabine	–
T. Leong 2017 (21)	RCT	120(60/60)	45/15		46/14	58 ± 13	56 ± 13		(Epirubicin + cisplatin + 5-flfluorouracil/capecitabine) + 45 Gy concurrently with 5-flfluorouracil/capecitabine	Epirubicin + cisplatin + 5-flfluorouracil/capecitabine	–
M. Stahl 2017 (22)	RCT	119(60/59)	54/6		54/5	Median age 60.6	Median age 56		5-flfluorouracil + folinic acid + cisplatin + 30 Gy with cisplatin and etoposide	5-flfluorouracil + folinic acid + cisplatin	–
X. Wang 2022 (7)	RCT	75(38/37)	31/7		30/7	18-75	18-75		40.04–45.1 Gy concurrently with S-1	SOX (S-1 + oxaliplatin)	–
Wang TB 2021 (16)	Retrospective	490 (100/390)	358/132			–	–		40.04 Gy concurrently with S-1	SOX (S-1 + oxaliplatin)	8
Li XH 2021 (23)	Retrospective	48(21/27)	15/6		19/8	–	–		XELOX(Oxaliplatin + capecitabine) + 46.8~50.4 Gy	XELOX(Oxaliplatin + capecitabine)	8
Fan GM 2018 (24)	Retrospective	89(44/45)	26/18		23/22	–	–		–	–	7
Zhang Y 2018 (25)	Retrospective	37(18/19)	–		–	62.56 ± 7.18	62.71 ± 4.79		TC(paclitaxel + carboplatin)+ 40 Gy	TC(paclitaxel + carboplatin)	8
Li J 2018 (26)	Retrospective	156(66/90)	61/5		72/18	–	–		45 Gy concurrently with XELOX(Oxaliplatin + capecitabine)	XELOX(Oxaliplatin + capecitabine)	8
C. C. Li 2022 (3)	Retrospective	63(38/25)	27/11		15/10	64	71		45-50.4Gy concurrently with mFOLFOX-4(5- flfluorouracil + folinic acid + oxaliplatin)	mFOLFOX-4(5- flfluorouracil + folinic acid + oxaliplatin)	8
Y. S. Yeh 2020 (27)	Retrospective	65(30/35)	22/8		20/15	–	–		45-50.4Gy concurrently with mFOLFOX-4(5- flfluorouracil + folinic acid + oxaliplatin)	mFOLFOX-4(5- flfluorouracil + folinic acid + oxaliplatin)	8
D. A. Trumbull 2021 (28)	Retrospective	413(329/84)	276/53		70/14	62.95 ± 9.97	63.88 ± 9.96		–	–	6
B.Azab 2019 (29)	Retrospective	4204 (2606/159)	–		–	–	–		–	–	6
E. L. Vos F. 2021 (30)	Retrospective	775(650/125)	(553/97)		(90/35)	63 (57-70)	62(55-68)		FLOT(5- flfluorouracil + folinic acid + oxaliplatin + docetaxel) + 50.4 Gy	FLOT(5- flfluorouracil + folinic acid + oxaliplatin + docetaxel)	8

### Quality assessment

**Figure 2:**
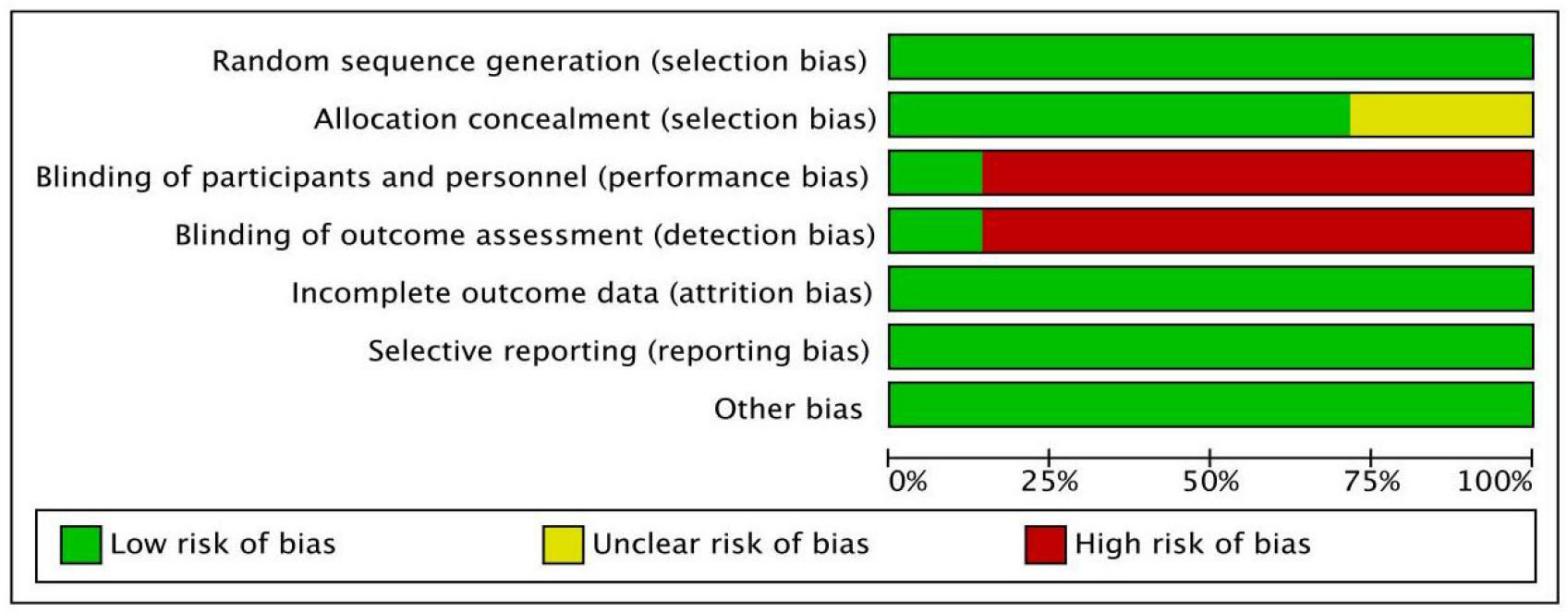
Risk of bias assessment for 7 RCTs.

**Figure 3:**
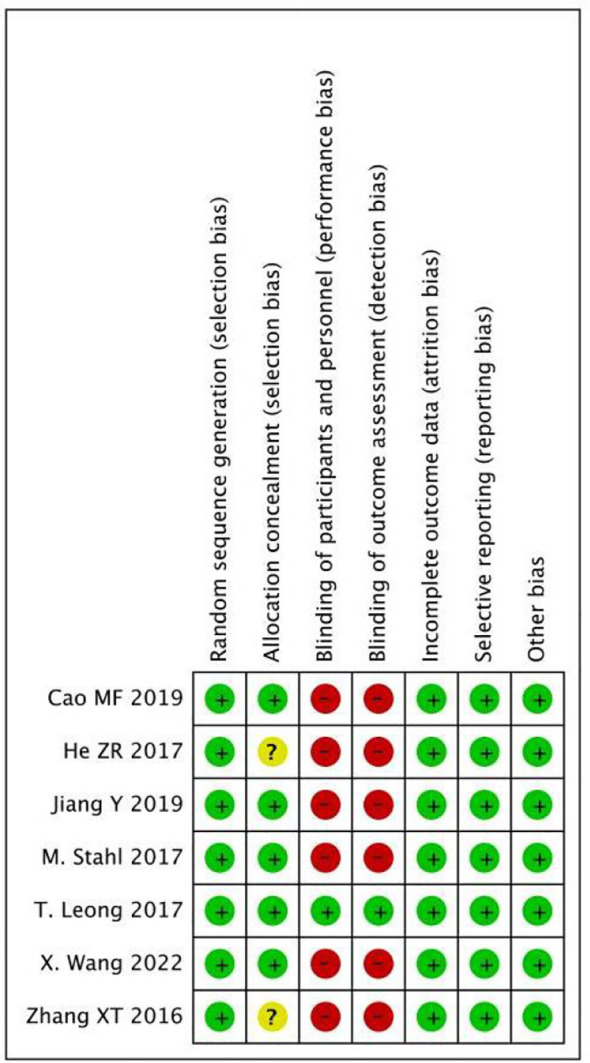
Risk of bias summary for 7 RCTs.

As shown in **Figures 2** and **3**, we evaluated the quality of the seven included RCTs using the Cochrane Collaboration’s tool, including randomsequence generation (selection bias), allocation concealment (selection bias), blinding of participants and personnel (performance bias), incomplete outcome data (attrition bias), selective reporting (reporting bias) and other biases, and assessing each risk of bias as high, low or unclear risk. We used the Review Manager software to graph and evaluate the results. Any disagreements arising from the process of data extraction and quality assessment were discussed and resolved by mutual agreement between the researchers. At the same time, the NOS was used to evaluate the retrospective studies and the scores were all greater than or equal to 6. The evaluation results are shown in **Table 1**. Overall, all the studies included in this meta - analysis were considered to be of high quality.

### Efficacy

#### CR analysis

**Figure 4:**
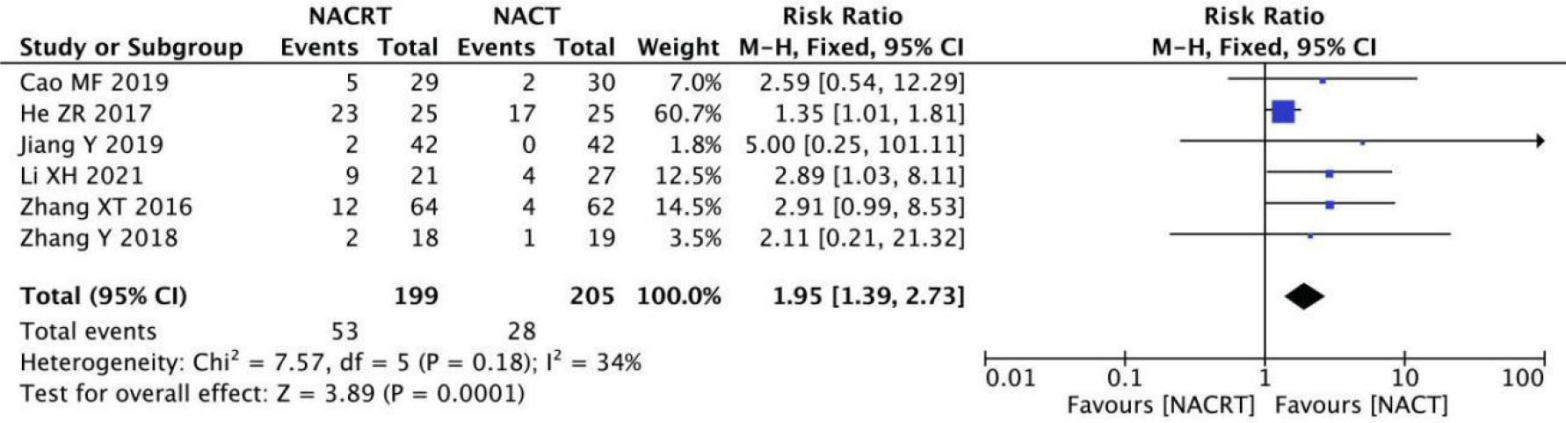
Forest plot for the analysis of the complete response CR.

CR was reported in 6 of the included studies, and the heterogeneity among the studies was not statistically significant (I^2 =^ 34%, P=0.18). Therefore, fixed effects were selected for meta-analysis. The results showed that CR in the NACRT group was higher than that in the NACT group (RR=1.95, 95%CI 1.39-2.73, P=0.0001< 0.05) (**Figure 4**).

#### PR analysis

**Figure 5:**
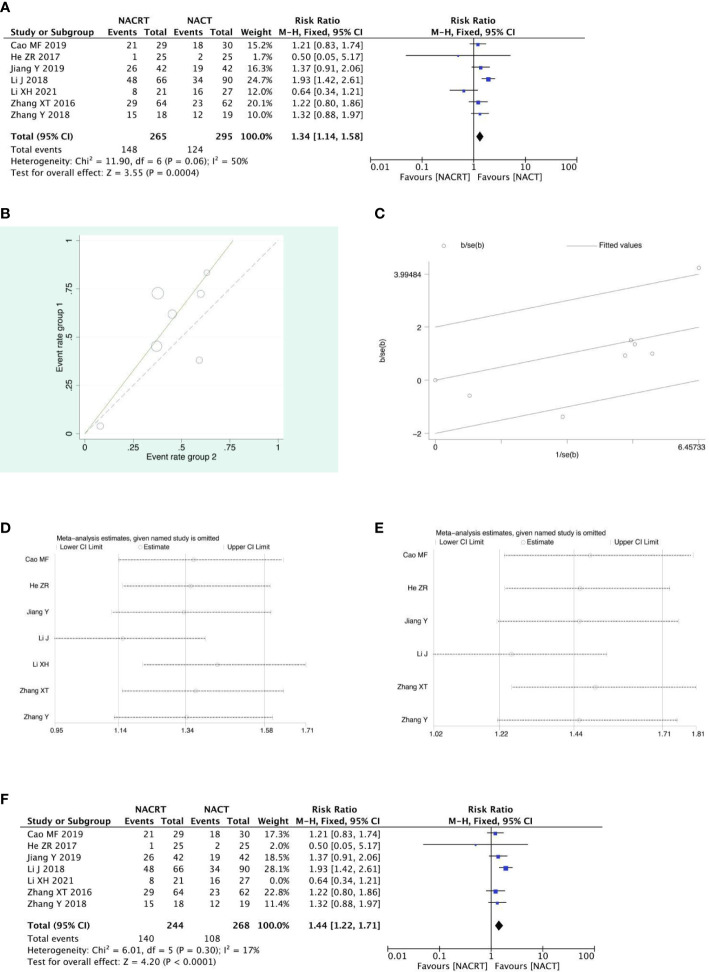
Forest plot **(A)**, Labet plot **(B)**, star plot **(C)**, first sensitivity analysis **(D)**, second sensitivity analysis **(E)**, forest plot after the second sensitivity analysis **(F)** for the analysis of the PR.

PR was reported in 7 of the included studies, and after heterogeneity testing, it was suggested that the heterogeneity between the selected literature in this study was statistically significant (I^2 =^ 50%, P=0.06) (**Figure 5A**), and after further investigation of the Labet plot (**Figure 5B**) and star chart (**Figure 5C**), it was found that one document may have strong heterogeneity and it is necessary to search for heterogeneity: we conducted a sensitivity analysis on the 7 studies this time and found that Li XH’s study had a greater impact on heterogeneity(**Figure 5D**), and the heterogeneity test was carried out again after removing the study. The results showed no heterogeneity in the remaining six studies (I^2 =^ 17%, P=0.3). Sensitivity analysis of the remaining studies was conducted again, and the results showed relatively stable (**Figure 5E**). Then, fixed effects were used for meta-analysisand after exclusion. The results showed that the PR of the NACRT group was higher than that in the NACT group (RR=1.44, 95% CI 1.22-1.71, P=0.0001<0.05) (**Figure 5F**).

#### SD analysis

**Figure 6:**
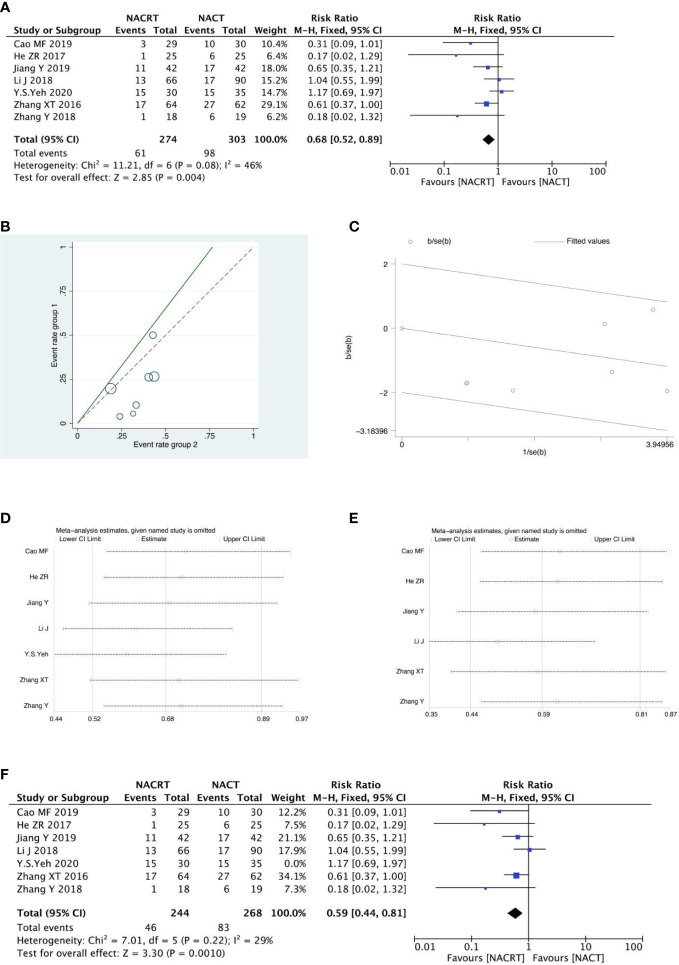
Forest plot **(A)**, Labet plot **(B)**, star plot **(C)**, first sensitivity analysis **(D)**, second sensitivity analysis **(E)**, forest plot after the second sensitivity analysis **(F)** for the analysis of the SD.

SD was reported in 7 of the included studies, and the heterogeneity test indicated that the heterogeneity among the literatures selected in this study was statistically significant (I^2 =^ 46%, P=0.08) (**Figure 6A**). After further investigation of Labet Chart (**Figure 6B**) and star chart (**Figure 6C**), it was found that the heterogeneity of one of the studies may be strong. Therefore, heterogeneity search should be carried out: we conducted sensitivity analysis on the 7 studies, and found that Y.S.EY’s study had a significant impact on heterogeneity (**Figure 6D**). After removing this study, we conducted heterogeneity test again, and the results showed that there was no heterogeneity in the remaining 6 studies (I^2 =^ 29, P=0.22). Sensitivity analysis of the remaining studies was conducted again, and the results showed relatively stable(**Figure 6E**). Then, fixed effect was selected for meta-analysis. The results showed that SD in the NACT group was higher than that in the NACRT group (RR=0.59, 95%CI 0.44-0.81, P=0.0010 <0.05) (**Figure 6F**).

#### PD analysis

**Figure 7:**
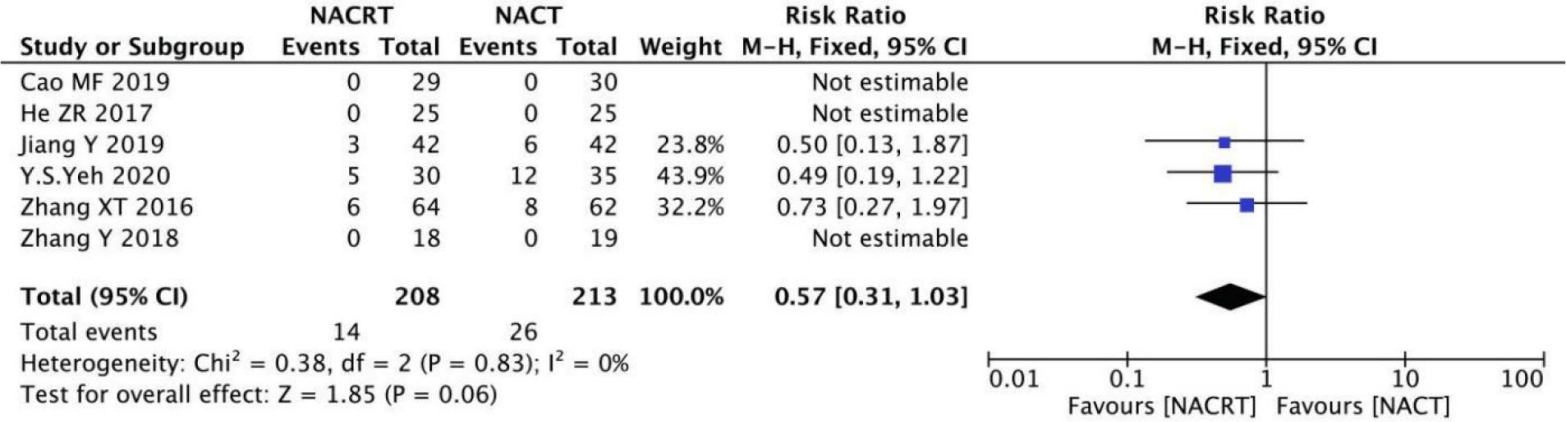
Forest plot for the analysis of the PD.

PD was reported in 6 of the included studies, and the heterogeneity among the studies was not statistically significant (I^2 =^ 0%, P=0.83). Therefore, fixed effects were selected for meta-analysis. The results showed that there was no significant difference in PD between the NACRT group and the NACT group (RR=0.57, 95%CI 0.31-1.03, P=0.06>0.05) (**Figure 7**).

#### ORR analysis

**Figure 8:**
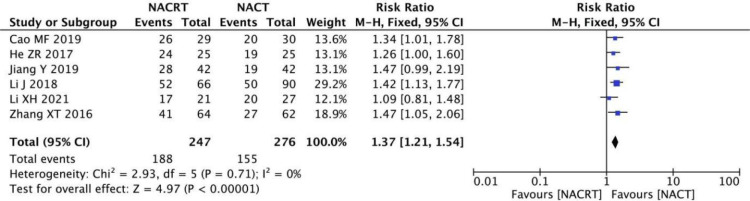
Forest plot for the analysis of the ORR.

ORR was reported in 6 of the included studies, and the heterogeneity among the studies was not statistically significant (I^2 =^ 0%, P=0.71). Therefore, fixed effects were selected for meta-analysis. The results showed that ORR in the NACRT group was higher than that in the NACT group (RR=1.37, 95%CI 1.27-1.54, P=0.00001<0.05) (**Figure 8**).

#### pCR rate analysis

**Figure 9:**
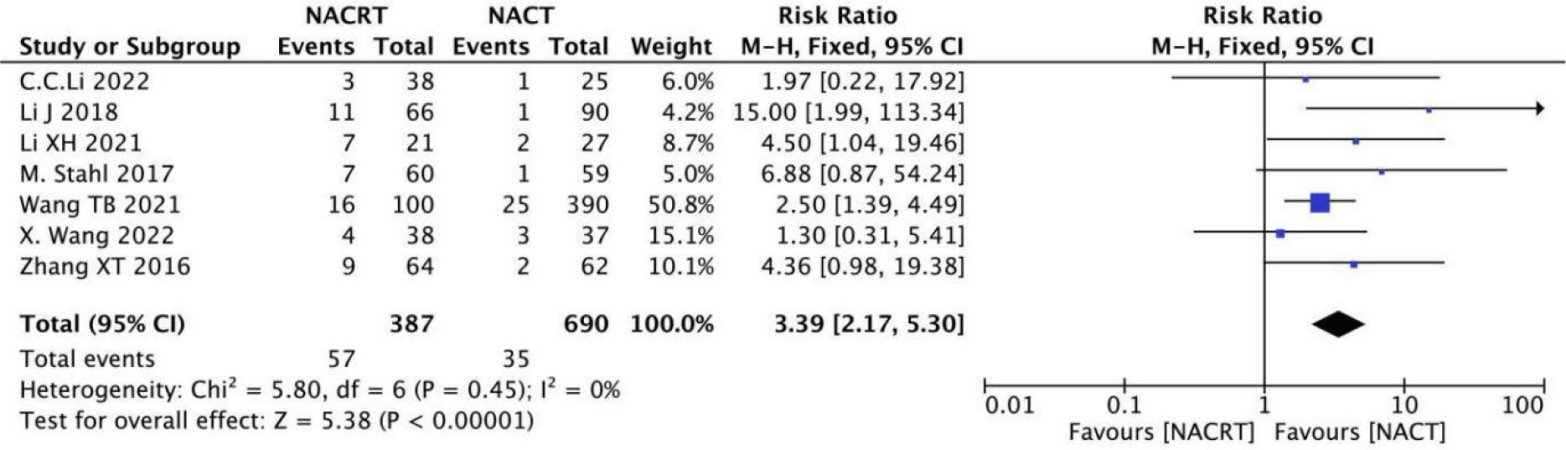
Forest plot for the analysis of the pCR.

pCR rate was reported in 7 of the included studies, and the heterogeneity among the studies was not statistically significant (I^2 =^ 0%, P=0.45). Therefore, fixed effects were selected for meta-analysis. The results showed that the pCR in the NACRT group was higher than that in the NACT group (RR=3.39, 95%CI 2.17-5.30, P=0.00001< 0.05) (**Figure 9**).

#### R0 resection rate analysis

**Figure 10:**
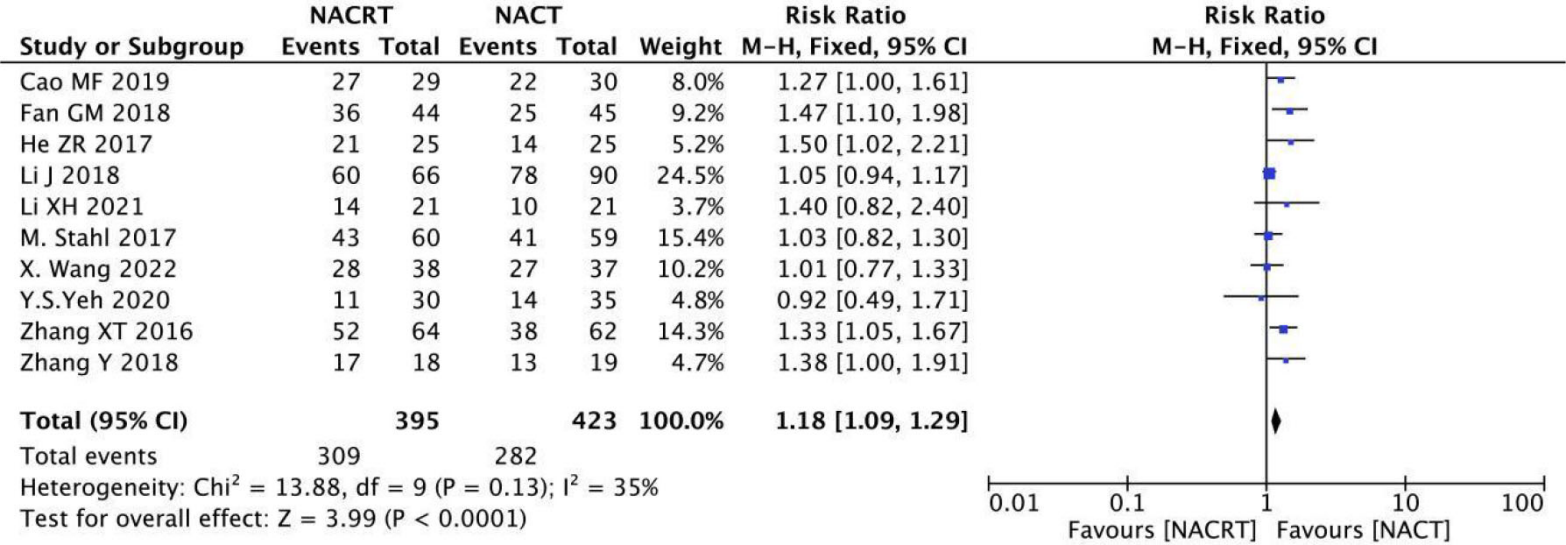
Forest plot for the analysis of the R0 resection rate.

R0 resection rate was reported in 10 of the included studies, and the heterogeneity among the studies was not statistically significant (I^2 =^ 35%, P=0.13). Therefore, fixed effects were selected for meta-analysis. The results showed that the R0 resection rate in the NACRT group was higher than that in the NACT group (RR=1.18, 95%CI 1.09-1.29, P=0.0001< 0.05) (**Figure 10**).

#### 3-year OS analysis

**Figure 11:**
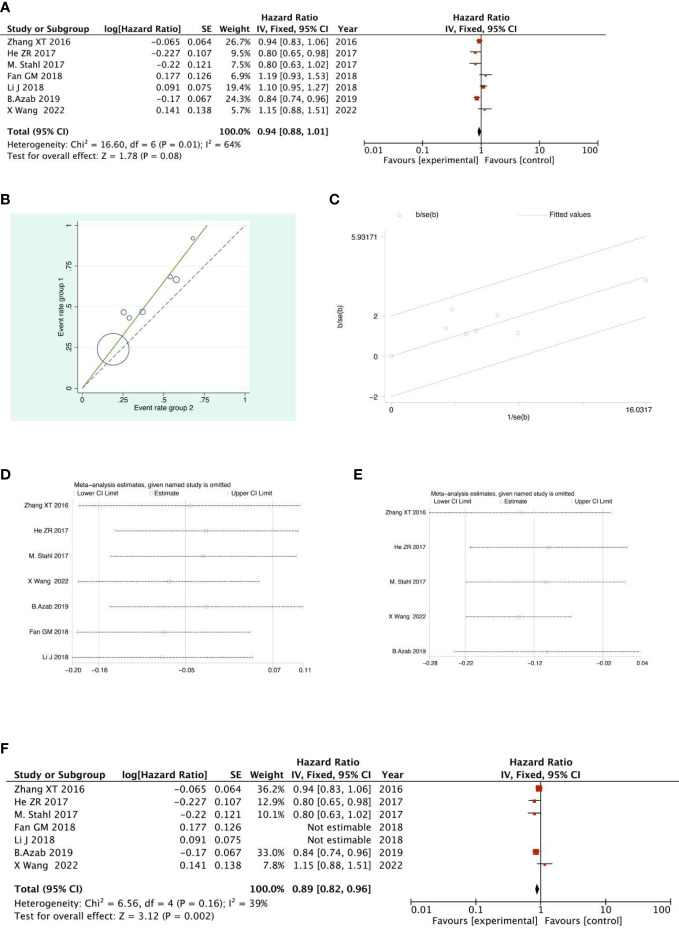
Forest plot **(A)**, Labet plot **(B)**, star plot **(C)**, first sensitivity analysis **(D)**, second sensitivity analysis **(E)**, forest plot after the second sensitivity analysis **(F)** for the analysis of the 3-year overall OS.

There were 4 RCTs and 3 retrospective studies of the included articles reporting 3-year OS. The results of the 3-year OS analysis showed that the heterogeneity test suggested that the heterogeneity among the included literatures was statistically significant (I^2 =^ 63.9%, P =0.011) (**Figure 11A**). After further investigation of Labet Chart (**Figure 11B**) and star chart (**Figure 11C**), it was found that the heterogeneity of two of the studies may be strong. Therefore, heterogeneity search should be carried out: we conducted sensitivity analysis on the 7 studies, and found that Fan GM’s study and Li J’s study had a significant impact on heterogeneity(**Figure 11D**). With those two studies removed, we conducted heterogeneity test again, and the results showed that there was no heterogeneity in the remaining 5 studies (I^2 =^ 38.9%, P=0.162). Sensitivity analysis of the remaining studies was conducted again, and the results showed relatively stable (**Figure 11E**). Then, fixed effect was selected for meta-analysis. The results showed that there was a significant difference in the 3-year OS between the NACRT group and the NACT group. Compared with the NACT group, NACRT could reduce the risk of death by 11% (HR=0.89, 95%CI 0.82-0.96, P=0.002<0.05) (**Figure 11F**).

#### 5-year OS analysis

**Figure 12:**
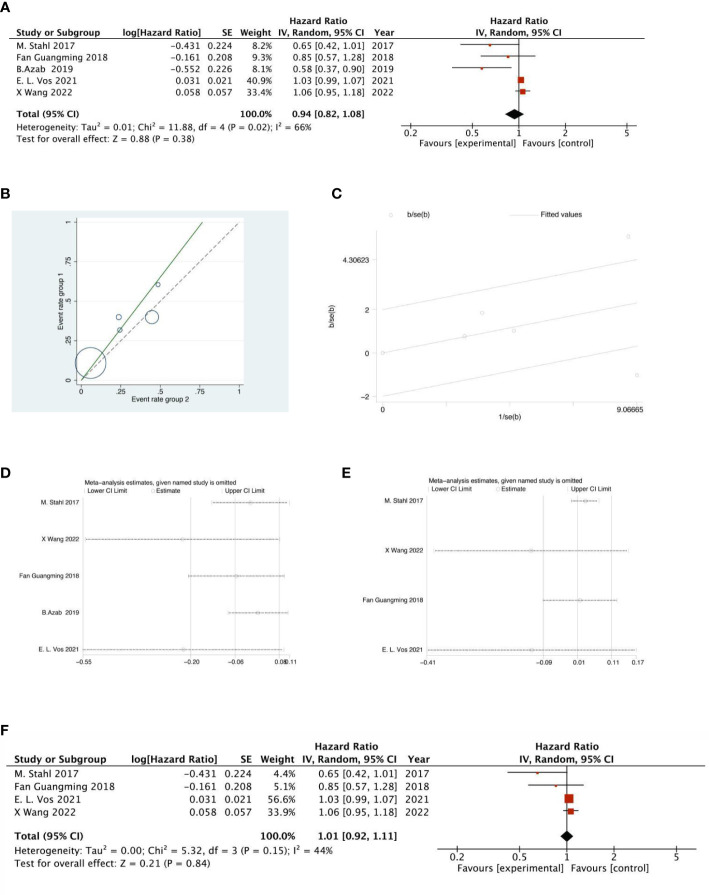
Forest plot **(A)**, Labet plot **(B)**, star plot **(C)**, first sensitivity analysis **(D)**, second sensitivity analysis **(E)**, forest plot after the second sensitivity analysis **(F)** for the analysis of the 5-year OS.

There were 2 RCTs and 3 retrospective studies of the included articles reporting 5-year OS. The analysis of the results of 5-year OS showed that the heterogeneity test indicated that the heterogeneity among the included literatures was statistically significant (I^2 =^ 66.4%, P =0.018) (**Figure 12A**). After further investigation of Labet Chart (**Figure 12B**) and star chart (**Figure 12C**), it was found that the heterogeneity of one of the studies may be strong. Therefore, heterogeneity search should be carried out: we conducted sensitivity analysis on the 5 studies, and found that B.Azab’s study had a significant impact on heterogeneity (**Figure 12D**). After removing this study, we conducted heterogeneity test again, and the results showed that there was no heterogeneity in the remaining 4 studies (I^2 =^ 43.6%, P=0.015). Sensitivity analysis of the remaining studies was conducted again, and the results showed relatively stable (**Figure 12E**). Then, fixed effect was selected for meta-analysis. The results showed that there was no significant difference in 5-year OS between NACRT group and NACT group (HR=1.03, 95%CI 0.99-1.07, P=0.839>0.05) (**Figure 12F**).

#### Subgroup analysis

After reviewing the each literature our analysis enrolled, we found that there was only one literature’s enrolled patients containing both GC and gastroesophageal junction cancer(GEJC) patients. Therefore, we differentiated the studies which containing GC and GEJC. The GC subgroup included eleven studies involving 5655 patients; the GEJC subgroup included five studies involving 1198 patients.

All subgroups demonstrated that ORR, pCR, R0 resection rate in the NACRT group were higher than that in the NACT group.The 3-year OS of the GC subgroup was higher in the NACRT group than that in the NACT group, while the GEJC subgroup showed no statistical difference between the two groups. The 5-year OS between the two subgroups was also not statistically significant. A comparison of outcomes is provided in **Table 2**, besides, more subgroup analysis results has been provided in the **Supplementary Materials**.

**Table 2:** Summary of surgical complications in included studies.

	Gastric cancer				Gastro-esophageal junction cancer			
	NO.	Rate (95% CI), %	I^2^, %	P value	NO.	Rate (95% CI), %	I^2^, %	P value
ORR	4	1.34 (1.13-1.59)	0	<.05	2	1.39 (1.17-1.66)	0	<.05
R0 resection	6	1.27 (1.10-1.46)	20	<.05	4	1.17 (1.06-1.29)	73	<.05
pCR	5	2.66 (1.66-4.27)	0	<.05	2	10.59 (2.55-43.94)	0	<.05
3-year OS	4	0.90 (0.83-0.97)	48	<.05	3	1.03 (0.92-1.15)	63	0.63
5-year OS	2	0.81 (0.45-1.47)	85	0.49	3	0.94 (0.82-1.08)	60	0.39

#### Adverse reactions to neoadjuvant therapy analysis

**Figure 13:**
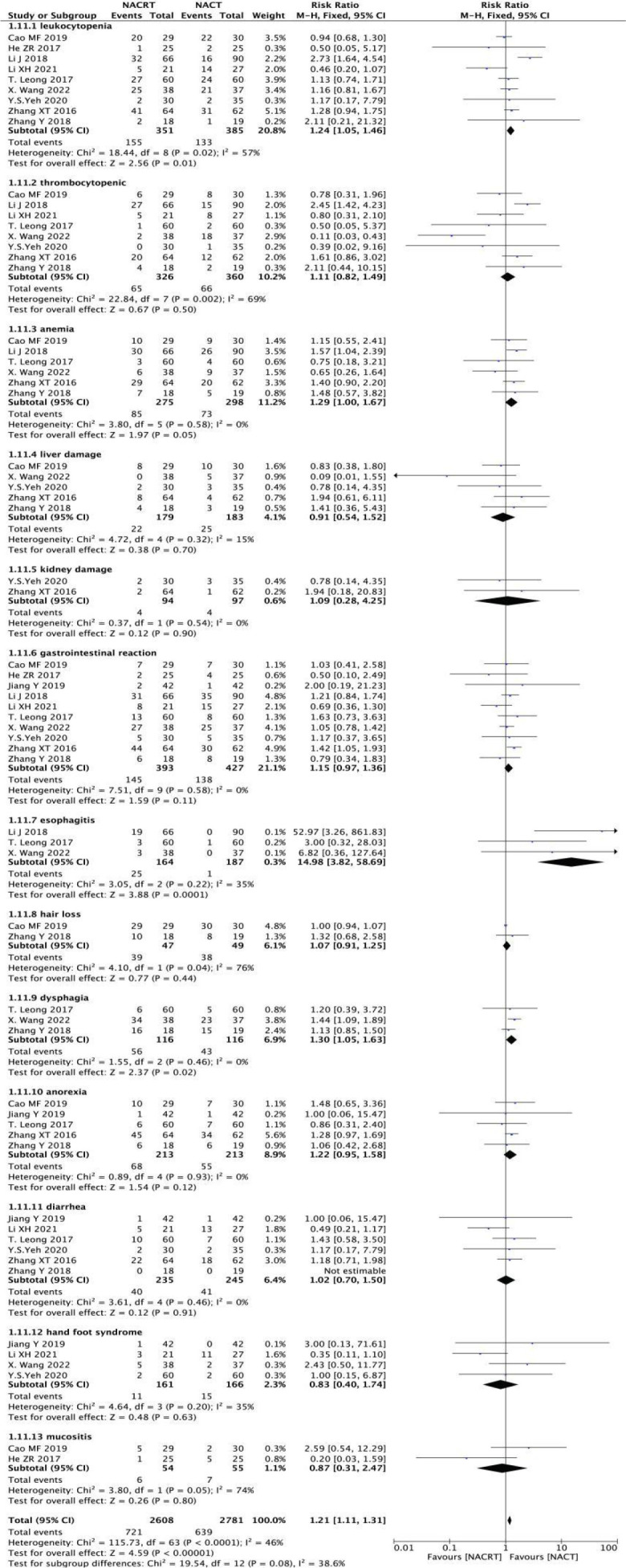
Forest plot for the analysis of the adverse reactions to neoadjuvant therapy.

In the included literature, nine studies reported leukopenia, eight studies reported thrombocytopenia, six studies reported anemia, five studies reported liver damage, two studies reported kidney damage, ten studies reported gastrointestinal reactions, three studies reported esophagitis, two studies reported hair loss, three studies reported dysphagia, three studies reported dysphagia, three studies reported dysphagia. Five studies reported anorexia, six studies reported diarrhea, four studies reported hand-foot syndrome and two studies reported mucosal inflammation. The incidence of esophagitis was higher in the NACRT group than in the NACT group (RR=14.98, 95%CI 3.82-58.69, P=0.0001< 0.05), there was no significant difference in other adverse reactions between the NACRT group and the NACT group, and the results were statistically significant (**Figure 13**).

#### Incidence of postoperative complications analysis

**Figure 14:**
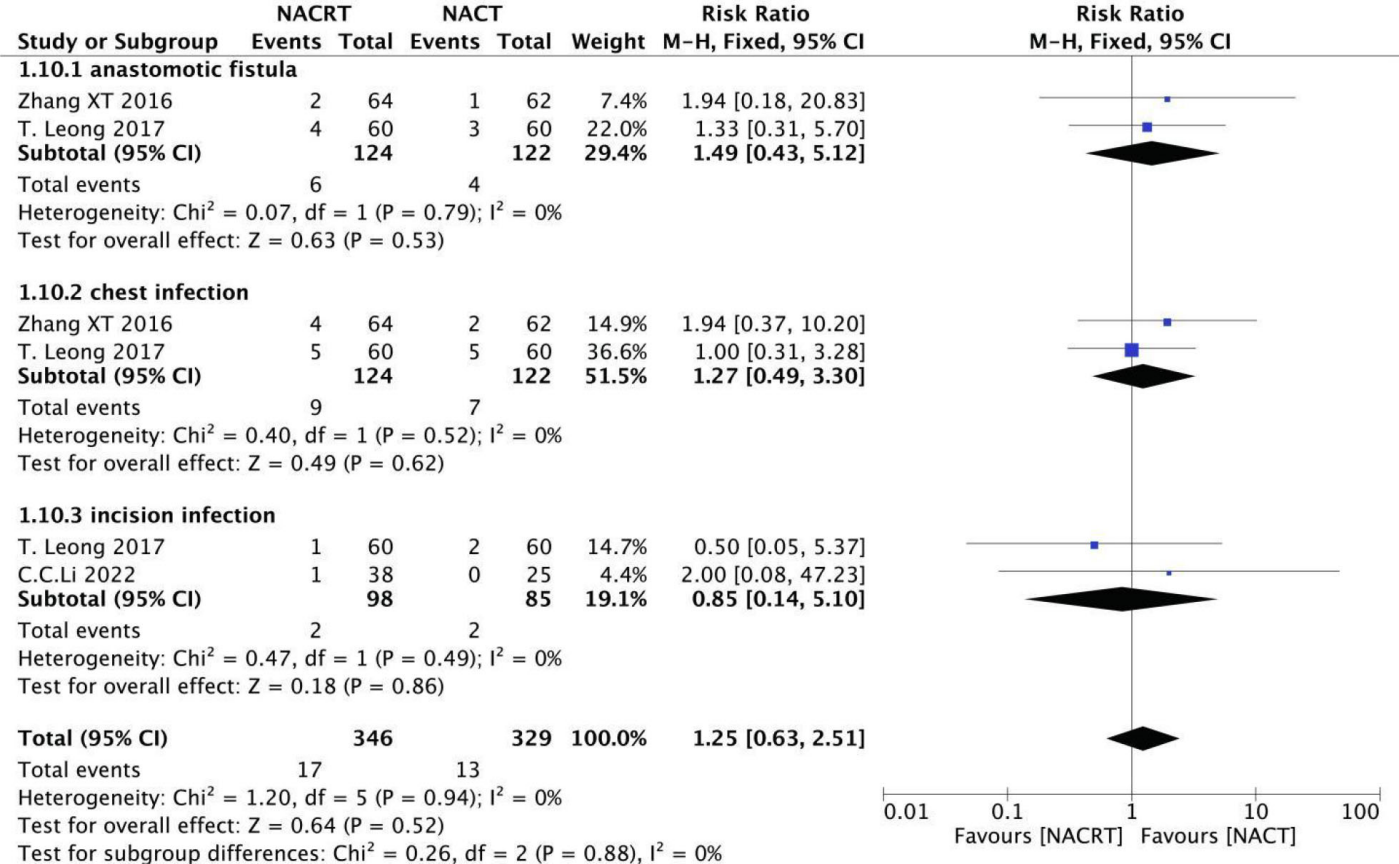
Forest plot for the analysis of the incidence of postoperative complications.

Among the included literatures, two reported anastomotic leakage (I^2 =^ 0%, P=0.79), two reported chest infection (I^2 =^ 0%, P=0.52), and two reported incision infection (I^2 =^ 0%, P=0.49), and the heterogeneity among the studies was not statistically significant. Therefore, We selected fixed effects for meta-analysis. The results showed that there was no significant difference in the incidence of postoperative complications between NACRT group and NACT group (**Figure 14**).

### Sensitivity and publication bias evaluation

**Figure 15:**
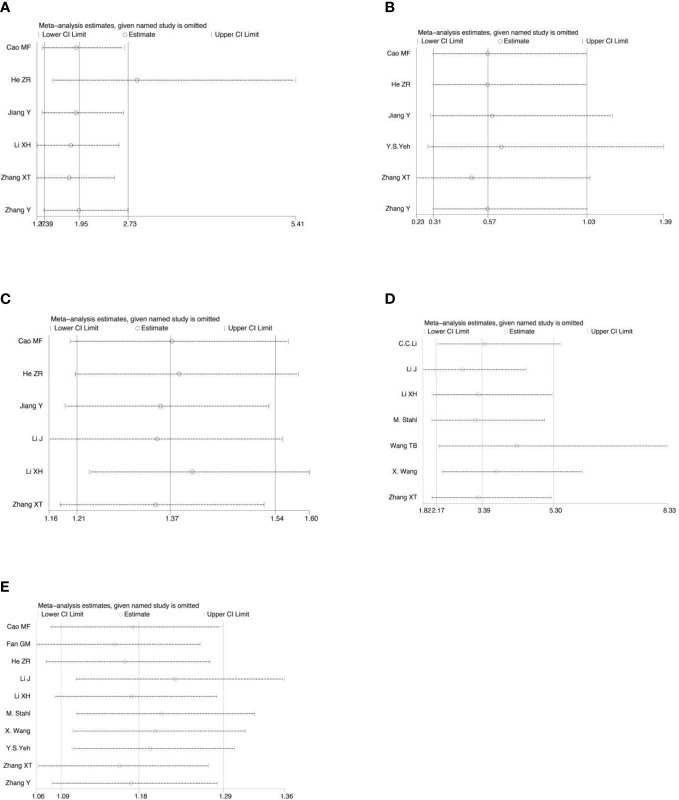
Sensitivity analysis for the analysis of CR **(A)**, PD **(B)**, ORR **(C)**, pCR rate **(D)** and R0 resection rate **(E)**.

**Figure 16:**
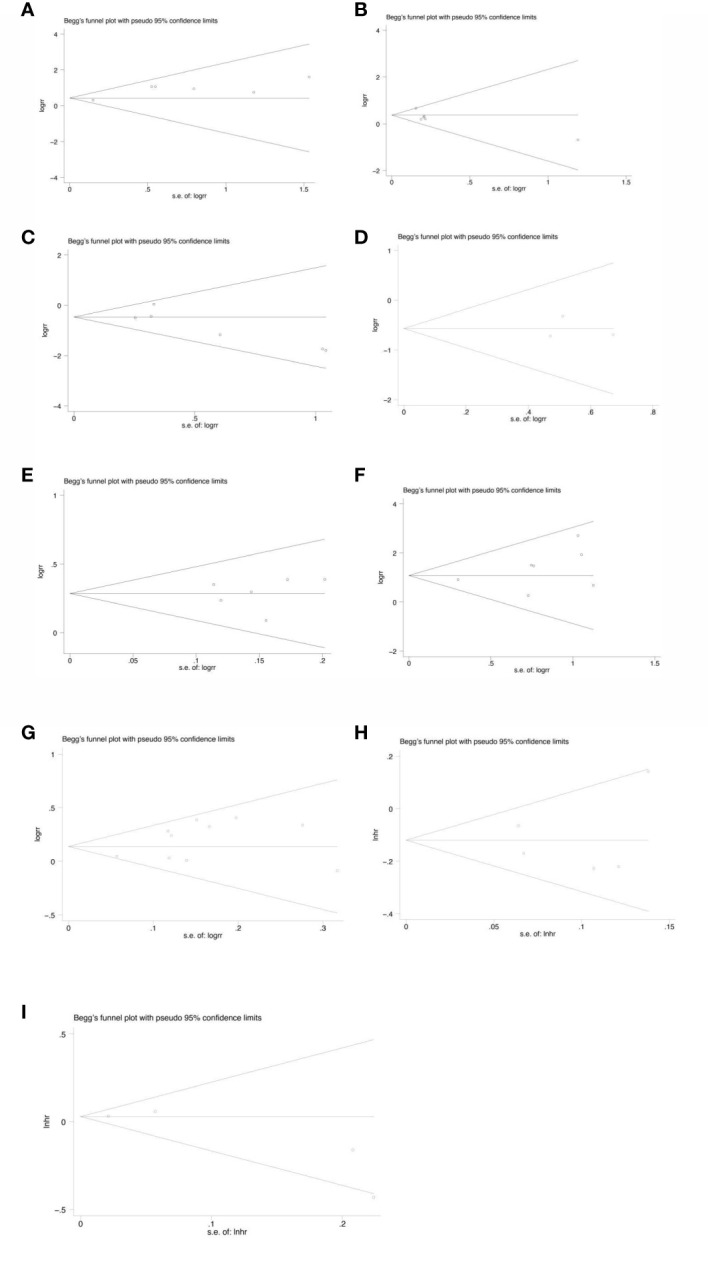
Begg’s funnel plot for the analysis of CR (p=1.000) **(A)**, PR(p=0.452) **(B)**, SD(p=0.260) **(C)**, PD(p=1.000) **(D)**, ORR(p=1.000) **(E)**, pCR rate(p=0.368) **(F)**, R0 resection rate(p=0.592) **(G)**, 3-year OS(p=0.221) **(H)**, 5-year OS(p=0.806) **(I)**.

Sensitivity analysis was performed on all the included outcome indicators, that is, deleting one study at a time to assess the impact of each study on the overall population. The results of meta-analysis are relatively stable (**Figure 15**). Begger funnel plot (**Figure 16**)were used to conduct bias test for the included outcome indicators, and the results showed that the removal rates of CR, PR, SD, PD, pCR, ORR, R0 resection rate, 3-year and 5-year OS had no significant publication bias.

## Discussion

GC is one of the most common malignant tumors in the world and the third most common cause of cancer death. Its incidence and mortality rank the second among all kinds of malignant tumors, second only to lung cancer. The 5-year survival rate is only 5%-15%. Since radical resection of LAGC is not feasible, clinical researchers have been seeking neoadjuvant methods to shrink the tumor in order to achieve the purpose of radical resection. Neoadjuvant therapy could increase the possibility of multimodal combined therapy, especially when surgical treatment is associated with serious complications and may hinder timely adjuvant therapy (27). The results of the CROSS trial indicated that neoadjuvant therapy reduced the risk of death from GC compared with surgery alone (HR=0.60, 95%CI 0.46-0.80) and the 10-year absolute OS benefit was 13% (38% vs 25%) (31).

NACRT and NACT are the standard therapies for late stage resection of GC (4). NACT is well tolerated by patients, which could reduce the clinical staging of tumors, create favorable conditions for surgery, inhibit the growth of small lesions, reduce the recurrence rate after surgery and increase the survival time. The study of ZHENG et al. have demonstrated the efficacy of NACT compared to surgery alone in LAGC patients (32). We speculate that the survival rate of patients after NACT may be increased due to the systemic response of chemotherapy. However, although NACT can prolong the survival of patients with LAGC, the extension time is limited. Most patients still have recurrence or metastasis, and the 5-year OS rate is less than 40% (33). At present, more and more researchers try to combine NACT with radiotherapy in order to further improve the survival rate of patients with LAGC. Nevertheless, the optimal mode of neoadjuvant therapy for LAGC patients is still controversial. Therefore, it is necessary to conduct a meta-analysis based on relevant RCT studies and retrospective studies to further explore the effectiveness and safety of NACRT in the treatment of LAGC.

This systematic review and meta-analysis included 7 RCTs and 9 retrospective studies. Our results showed that NACRT improved ORR, R0 resection rate and pCR rate and patients had more favorable 3-year OS and even an 11% reduced risk of death but there was no significant improvement in 5-year OS. Actually, most GC often recurred at distant sites, more specifically, peritoneal implantation metastasis rather than local recurrence (28). As a systemic therapy, systemic chemotherapy at the early stage of metastasis is its advantage but our meta-analysis showed that NACRT at the early stage can better improve patients’ survival. In terms of toxicity and side effects, the incidence of esophagitis in the NACRT group was higher than that in the NACT group and there was no significant difference in other adverse reactions between the experimental group and the control group, which confirmed that NACRT was safe and effective, and the adverse reactions were controllable.

The results of our meta-analysis showed that NACRT could bring more favorable OS to patients with LAGC compared with NACT. Although Y. S. Yeh et al. (27), C. C. Li et al. (3) and some other studies have proved the benefits of NACRT on OS for LAGC patients in recent years, some studies have reached contrary conclusions to our study. The results of many previous studies suggest that although NACRT group could bring higher pCR rate and down-time rate, it cannot be converted into survival advantage. Denslow A. Trumbull et al. (28) found that compared with NACT group, patients receiving NACRT can obtain higher pCR rate but that didn’t translate into better OS. The 5-year OS rate of GC patients was only 60%, while that of NACT group was 90%. A study of E. L. Vos et al. (30) also mentioned that although patients in NACRT group achieved better descending effect, it did not bring better survival for patients. In their research, although the tumors and lymph nodes in the NACRT group showed significant pathological downphase after treatment, there was no significant difference in OS or DFS at 3 years (22, 34). In addition, radiotherapy has unique toxicity to the target tissue, which may lead to increased difficulty of hand resection or postoperative complications, all of which will affect the survival of patients.

However, our meta-analysis also has some limitations. For example, most of the included studies did not carry out long-term follow-up, and the results of OS were rarely reported. In addition, there were differences in the level of GC surgeons, the time interval between neoadjuvant therapy and surgical treatment, radiation dose, chemotherapy regimen and dose administration among the studies. Whatsmore, there have been an important ongoing trial TOPGEAR (21) which is assessing NACRT vs NACT in LAGC patients, however, it only reported the interim results regarding adverse effects after neoadjuvant therapy and postoperative complications: grade 3 or higher gastrointestinal toxicity occurred in 30% (NACRT group) and 32% (NACT group) of patients, while hematologic toxicity occurred in 52% and 50%. Furthermore, grade 3 or higher surgical complications occurred in 22% of patients in both groups. These results demonstrate that NACRT can be safely delivered to the vast majority of patients without a signifificant increase in treatment toxicity or surgical morbidity. Unfortunately, since the trial is ongoing, many therapeutic indicators such as pCR rate and OS are not avaliable. Whereas we expected the final results of this trial and we believe that it will help our research. Finally, FLOT regimen is currently a Class I recommendation of perioperative chemotherapy for GC as recommended by NCCN guidelines, none of the studies included (with the exception of the study by Vos in GEJC) use this regimen as NACT, although other included studuies didn’t use FLOT protocol but all used the regimen which the guidelines recommended. All of these factors may affect the results of our meta-analysis.

To sum up, OS is still controversial in patients with LAGC, and we look forward to including more qualified cases and longer follow-up to make the conclusions more robust.

## Conclusion

In conclusion, compared with NACT, NACRT improved the ORR, R0 resection rate and pCR rate, and patients obtained more favorable OS, and there was no significant increase in toxic side effects. Therefore, we can conclude that compared with preoperative chemotherapy alone, NACRT may be a safer and more effective regimen in the treatment of LAGC patients. There are still a number of phase three clinical trials underway, and we look forward to more results to demonstrate the effectiveness of NACRT.

## Data availability statement

The original contributions presented in the study are included in the article/**Supplementary Material**. Further inquiries can be directed to the corresponding authors.

## Author contributions

YZ and JC have made equal contributions to this article. All authors contributed to the article and approved the submitted version.

## References

[1] Machlowska J Baj J Sitarz M Maciejewski R Sitarz R . Gastric cancer: epidemiology, risk factors, classification, genomic characteristics and treatment strategies. Int J Mol Sci (2020) 21(11):2. doi: 10.3390/ijms21114012 PMC7312039 32512697

[2] Sung H Ferlay J Siegel RL Laversanne M Soerjomataram I Jemal A . Global cancer statistics 2020: GLOBOCAN estimates of incidence and mortality worldwide for 36 cancers in 185 countries. CA Cancer J Clin (2021) 71(3):209–49. doi: 10.3322/caac.21660 33538338

[3] Li CC Yeh YS Chen YC Su WC Chang TK Tsai HL . Surgical efficacy and safety of patients with locally advanced gastric cancer following neoadjuvant concurrent chemoradiotherapy and chemotherapy. J Oncol (2022) 2022:4. doi: 10.1155/2022/3719241 PMC8957450 35345514

[4] Cats A Jansen EPM van Grieken NCT Sikorska K Lind P Nordsmark M . Chemotherapy versus chemoradiotherapy after surgery and preoperative chemotherapy for resectable gastric cancer (CRITICS): an international, open-label, randomised phase 3 trial. Lancet Oncol (2018) 19(5):616–28. doi: 10.1016/S1470-2045(18)30132-3 29650363

[5] Hizal M Sendur MA Bilgin B Bulent Akinci M Sener Dede D Yalcin B . Expanding treatment options for resectable gastric cancer: is it a countdown for radiotherapy? J buon (2019) 24(4):1367–70. 31646779

[6] Ajani JA D’Amico TA Bentrem DJ Chao J Cooke D Corvera C . Gastric cancer, version 2.2022, NCCN clinical practice guidelines in oncology. J Natl Compr Canc Netw (2022) 20(2):167–92. doi: 10.6004/jnccn.2022.0008 35130500

[7] Wang X Zhao DB Yang L Chi Y Zhao H Jiang LM . Preoperative concurrent chemoradiotherapy versus neoadjuvant chemotherapy for locally advanced gastric cancer: phase II randomized study. Front Oncol (2022) 12. doi: 10.3389/fonc.2022.870741 PMC9104815 35574368

[8] Ajani JA Mansfield PF Crane CH Wu TT Lunagomez S Lynch PM . Paclitaxel-based chemoradiotherapy in localized gastric carcinoma: degree of pathologic response and not clinical parameters dictated patient outcome. J Clin Oncol (2005) 23(6):1237–44. doi: 10.1200/JCO.2005.01.305 15718321

[9] Ajani JA Mansfield PF Janjan N Morris J Pisters PW Lynch PM . Multi-institutional trial of preoperative chemoradiotherapy in patients with potentially resectable gastric carcinoma. J Clin Oncol (2004) 22(14):2774–80. doi: 10.1200/JCO.2004.01.015 15254045

[10] Liu X Li G Long Z Yin J Zhu X Sheng W . Phase II trial of preoperative chemoradiation plus perioperative SOX chemotherapy in patients with locally advanced gastric cancer. J Surg Oncol (2018) 117(4):692–8. doi: 10.1002/jso.24917 29194623

[11] Tian S Jiang R Madden NA Ferris MJ Buchwald ZS Xu KM . Survival outcomes in patients with gastric and gastroesophageal junction adenocarcinomas treated with perioperative chemotherapy with or without preoperative radiotherapy. Cancer (2020) 126(1):37–45. doi: 10.1002/cncr.32516 31532544

[12] Ikoma N Das P Hofstetter W Ajani JA Estrella JS Chen HC . Preoperative chemoradiation therapy induces primary-tumor complete response more frequently than chemotherapy alone in gastric cancer: analyses of the national cancer database 2006-2014 using propensity score matching. Gastric Cancer. (2018) 21(6):1004–13. doi: 10.1007/s10120-018-0832-z PMC6515902 29730720

[13] Petrelli F Ghidini M Barni S Sgroi G Passalacqua R Tomasello G . Neoadjuvant chemoradiotherapy or chemotherapy for gastroesophageal junction adenocarcinoma: a systematic review and meta-analysis. Gastric Cancer. (2019) 22(2):245–54. doi: 10.1007/s10120-018-0901-3 30483986

[14] Xiang M Chang DT Heestand GM Pollom EL . Survival after neoadjuvant approaches to gastroesophageal junction cancer. Gastric Cancer. (2020) 23(1):175–83. doi: 10.1007/s10120-019-00980-6 31230228

[15] Zafar SN Blum M Chiang YJ Ajani JA Estrella JS Das P . Preoperative chemoradiation versus chemotherapy in gastroesophageal junction adenocarcinoma. Ann Thorac Surg (2020) 110(2):398–405. doi: 10.1016/j.athoracsur.2020.03.024 32289300

[16] Wang TB Zhou H Zhang XJ Sun CY Guo CG Zhou AP . The influencing factors and prognosis analysis of neoadjuvant therapy for pathological complete response of gastric cancer. Acta Academiae Medicinae Sinicae (2021) 43(4):6. doi: 10.3881/j.issn.1000-503X.13260 34494528

[17] Zhang XT Zhang Z Liu L Xin YN Xuan SY . Comparative study of neoadjuvant therapy for locally advanced gastric cancer. Chin J Of Cancer Prev And Treat (2016) 23(11).

[18] Cao MF . Short-term efficacy and safety evaluation of paclitaxel carboplatin one-week chemotherapy regimen and concurrent radiotherapy regimen in neoadjuvant therapy for stage III esophageal and gastric conjunctive adenocarcinoma. Hebei, China: Hebei North University (2019).

[19] Jiang Y Jin S Shen Q . Preoperative intensity modulated radiotherapy combined with concurrent capecitabine chemotherapy for neoadjuvant chemoradiotherapy in advanced gastric cancer. China Foreign Med Treat (2019) 38(10).

[20] R HZ Gu WG Hu ZX Song HB . Effect of neoadjuvant chemoradiotherapy on locally advanced gastric cancer. Heilongjiang Med J (2017) 30(2). doi: 10.14035/j.cnki.hljyy.2017.02.006

[21] Leong T Smithers BM Haustermans K Michael M Gebski V Miller D . TOPGEAR: a randomized, phase III trial of perioperative ECF chemotherapy with or without preoperative chemoradiation for resectable gastric cancer: interim results from an international, intergroup trial of the AGITG, TROG, EORTC and CCTG. Ann Surg Oncol (2017) 24(8):2252–8. doi: 10.1245/s10434-017-5830-6 28337660

[22] Stahl M Walz MK Riera-Knorrenschild J Stuschke M Sandermann A Bitzer M . Preoperative chemotherapy versus chemoradiotherapy in locally advanced adenocarcinomas of the oesophagogastric junction (POET): long-term results of a controlled randomised trial. Eur J Cancer (Oxford England: 1990). (2017) 81:183–90. doi: 10.1016/j.ejca.2017.04.027 28628843

[23] Li XH Yu XJ Zhang J Yu YD Di QS . Comparison of efficacy and safety of neoadjuvant chemoradiotherapy and neoadjuvant chemoradiotherapy for advanced gastric cancer. CHONGQING Med (2021) 50(1). doi: 10.3969/j.issn.1671-8348.2021.01.015

[24] Fan GM Wang JQ Su PC . Comparison of neoadjuvant chemoradiotherapy and neoadjuvant chemoradiotherapy in the prognosis of locally advanced esophagogastric conjunctive adenocarcinoma. Proceeding Clin Med (2018) 27(7).

[25] Zhang Y . Evaluation of short-term efficacy and safety of neoadjuvant concurrent chemoradiotherapy for stage III cardiac cancer. Hebei, China: Hebei North University (2018).

[26] Li J . Comparison of efficacy of neoadjuvant chemoradiotherapy and neoadjuvant chemoradiotherapy in siewert type II and III locally advanced esophagogastric junction adenocarcinoma [Master’s degree]. Hebei, China: Hebei Medical University (2018).

[27] Yeh YS Huang MY Ma CJ Huang CW Tsai HL Chen YC . Observational study comparing efficacy and safety between neoadjuvant concurrent chemoradiotherapy and chemotherapy for patients with unresectable locally advanced or metastatic gastric cancer. J Oncol (2020) 2020:7. doi: 10.1155/2020/6931317 PMC7492932 32963531

[28] Trumbull DA Lemini R Díaz Vico T Jorgensen MS Attwood K Ji W . Prognostic significance of complete pathologic response obtained with chemotherapy versus chemoradiotherapy in gastric cancer. Ann Surg Oncol (2021) 28(2):766–73. doi: 10.1245/s10434-020-08921-9 32737698

[29] Azab B Macedo F Picado O Franceschi D Livingstone AS Yakoub D . The impact of neoadjuvant chemoradiation versus chemotherapy on short and long-term outcomes among gastric carcinoma patients. Ann Surg Oncol (2019) 26:S60–. doi: 10.1245/s10434-018-6897-4 30311162

[30] Vos EL Carr RA Hsu M Nakauchi M Nobel T Russo A . Prognosis after neoadjuvant chemoradiation or chemotherapy for locally advanced gastro-oesophageal junctional adenocarcinoma. Br J Surg (2021) 108(11):1332–40. doi: 10.1093/bjs/znab228 PMC8599637 34476473

[31] Eyck BM van Lanschot JJB Hulshof M van der Wilk BJ Shapiro J van Hagen P . Ten-year outcome of neoadjuvant chemoradiotherapy plus surgery for esophageal cancer: the randomized controlled CROSS trial. J Clin Oncol (2021) 39(18):1995–2004. doi: 10.1200/JCO.20.03614 33891478

[32] Zheng CH Lu J Huang CM Li P Xie JW Wang JB . Treatment of locally advanced gastric cancer with the XELOX program of neoadjuvantchemotherapy combined with laparoscopic surgery: the experience in China. Hepatogastroenterology (2014) 61(135):1876–82. 25713882

[33] Chen J Ye Q Huang F . [Historical evolution and research progress of perioperative therapy of locally advanced gastric cancer]. Zhonghua Wei Chang Wai Ke Za Zhi. (2019) 22(2):196–200. 30799543

[34] Spicer JD Stiles BM Sudarshan M Correa AM Ferri LE Altorki NK . Preoperative chemoradiation therapy versus chemotherapy in patients undergoing modified en bloc esophagectomy for locally advanced esophageal adenocarcinoma: is radiotherapy beneficial? Ann Thorac Surg (2016) 101(4):1262–9; discussion 969-70. doi: 10.1016/j.athoracsur.2015.11.070 26916717

