# Supplementary material for: Survival and complications after neoadjuvant chemoradiotherapy versus neoadjuvant chemotherapy for locally advanced gastric cancer: a systematic review and meta-analysis
Source: Front Oncol. 2023 May 9;13:1177557. doi: 10.3389/fonc.2023.1177557 (PMC10203550; doi:10.3389/fonc.2023.1177557)
Supplement: Supplementary file 1 [file DataSheet_1.docx]

# Supplementary materials caption

**Figure S1：**Funnel plot(A), Egger’s plot(B), Begg’s and Egger’s tests(C) for publication bias for the analysis of the Complete response(CR).

**Figure S2：**Funnel plot(A), Funnel plot after the second sensitivity analysis(B),Egger’s plot(C), Begg’s and Egger’s tests(D) for publication bias for the analysis of the partial response(PR).

**Figure S3：**Funnel plot(A), Funnel plot after the second sensitivity analysis(B),Egger’s plot(C), Begg’s and Egger’s tests(D) for publication bias for the analysis of the stable disease(SD).

**Figure S4：**Funnel plot(A), Egger’s plot(B), Begg’s and Egger’s tests(C) for publication bias for the analysis of the progressive disease(PD).

**Figure S5：**Funnel plot(A), Egger’s plot(B), Begg’s and Egger’s tests(C) for publication bias for the analysis of the objective response rate(ORR).

**Figure S6：**Funnel plot(A), Egger’s plot(B), Begg’s and Egger’s tests(C) for publication bias for the analysis of the pathologic complete response(pCR).

**Figure S7：**Funnel plot(A), Egger’s plot(B), Begg’s and Egger’s tests(C) for publication bias for the analysis of the R0 resection rate.

**Figure S8：**Funnel plot(A), Funnel plot after the second sensitivity analysis(B),Egger’s plot(C), Begg’s and Egger’s tests(D) for publication bias for the analysis of the 3-year overall survival(OS).

**Figure S9：**Funnel plot(A), Funnel plot after the second sensitivity analysis(B),Egger’s plot(C), Begg’s and Egger’s tests(D) for publication bias for the analysis of the 5-year OS.

**Figure S10：**Forest plot(A) and Funnel plot(B) for the subgroup analysis of the ORR.

**Figure S11：**Forest plot(A) and Funnel plot(B) for the subgroup analysis of the pCR.

**Figure S12：**Forest plot(A) and Funnel plot(B) for the subgroup analysis of the R0 resection rate.

**Figure S13：**Forest plot(A) and Funnel plot(B) for the subgroup analysis of the 3-year OS.

**Figure S14：**Forest plot(A) and Funnel plot(B) for the subgroup analysis of the 5-year OS.

A B

| 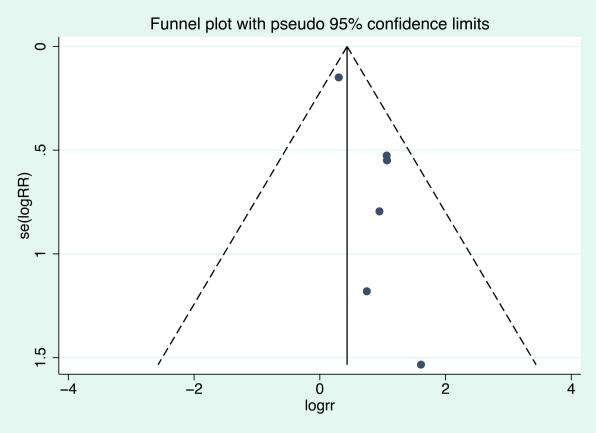 | 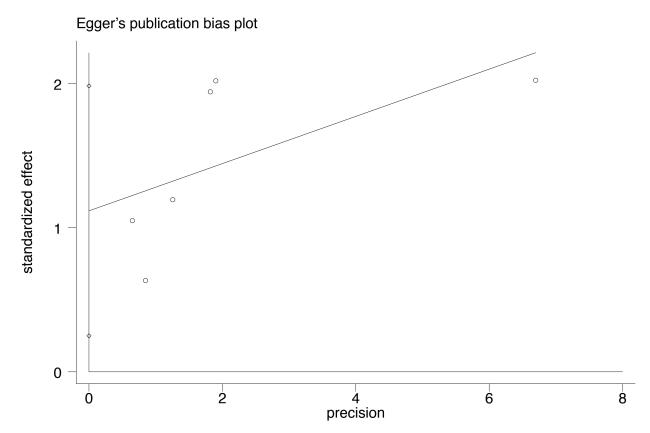 |
| --- | --- |

C
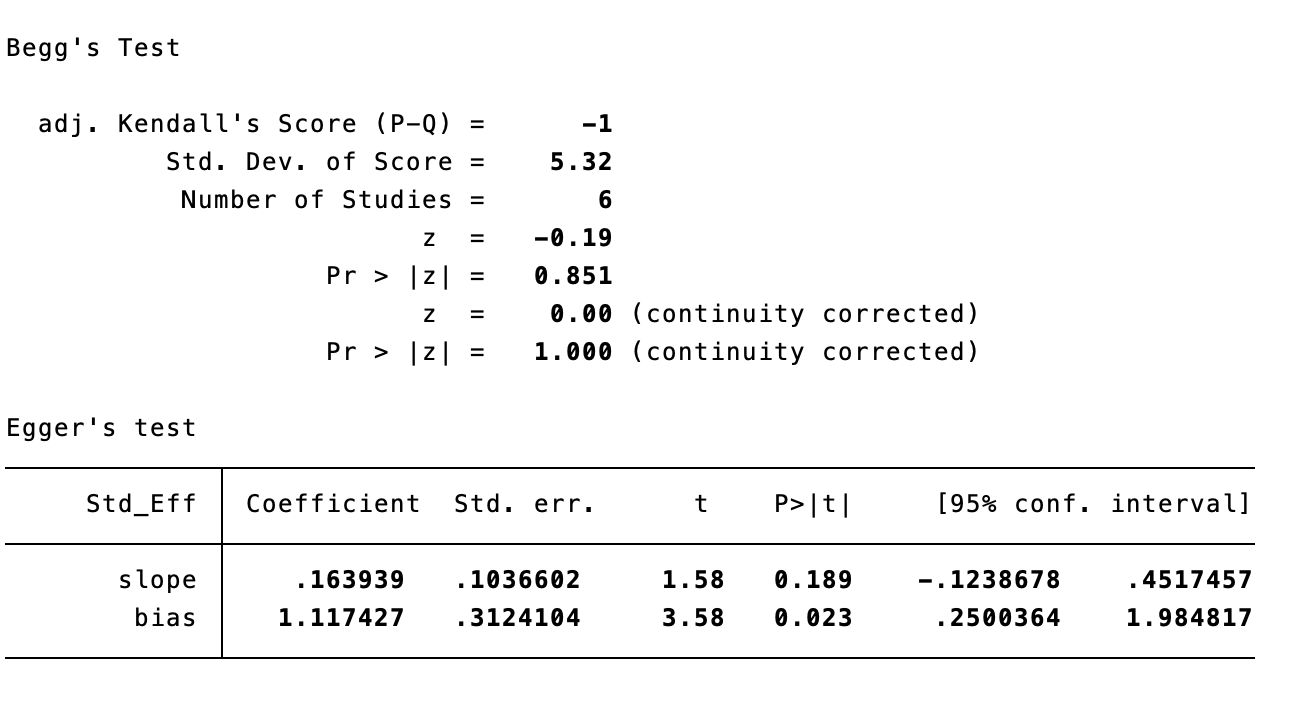


**Figure S1：**Funnel plot(A), Egger’s plot(B), Begg’s and Egger’s tests(C) for publication bias for the analysis of the CR.

A B

| 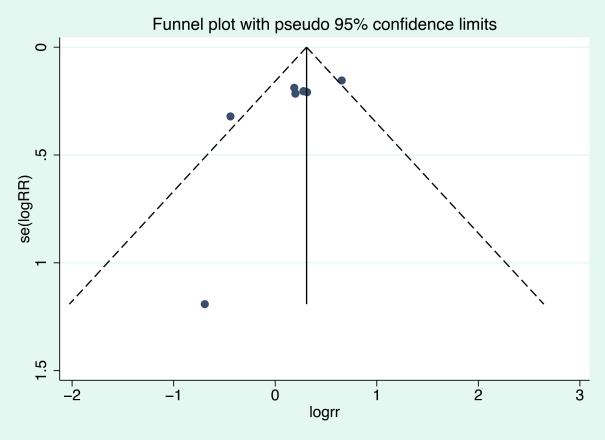 | 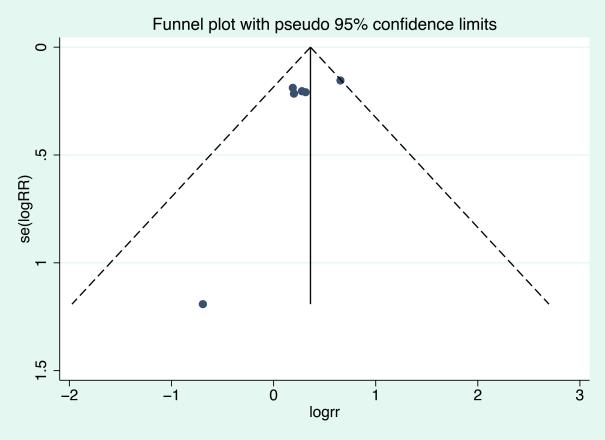 |
| --- | --- |

C


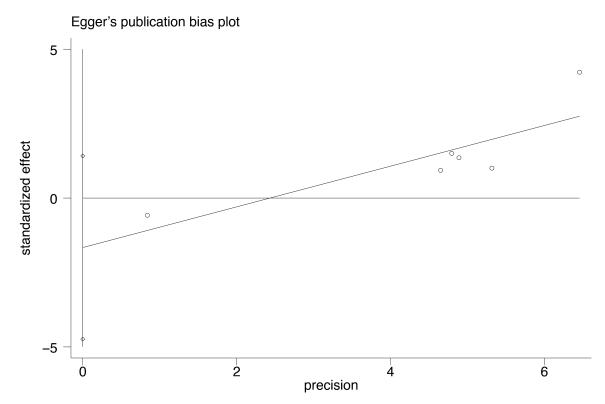


D


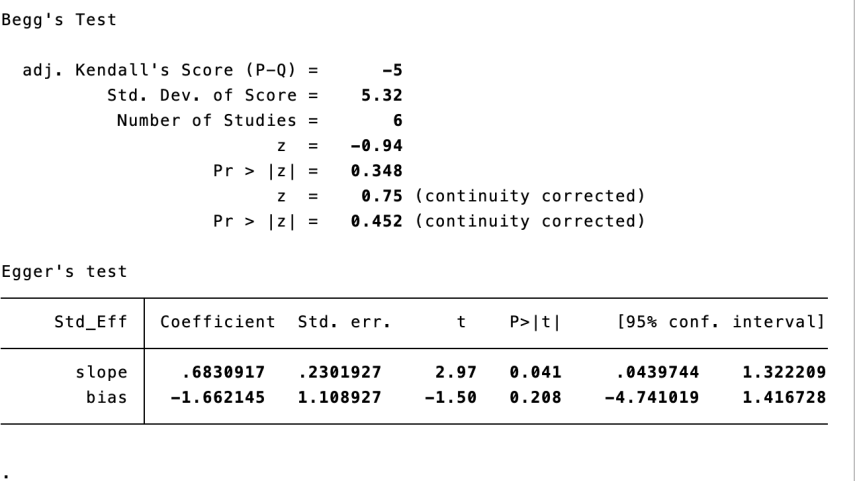


**Figure S2:** Funnel plot(A), Funnel plot after the second sensitivity analysis(B),Egger’s plot(C), Begg’s and Egger’s tests(D) for publication bias for the analysis of the PR.

A B

| 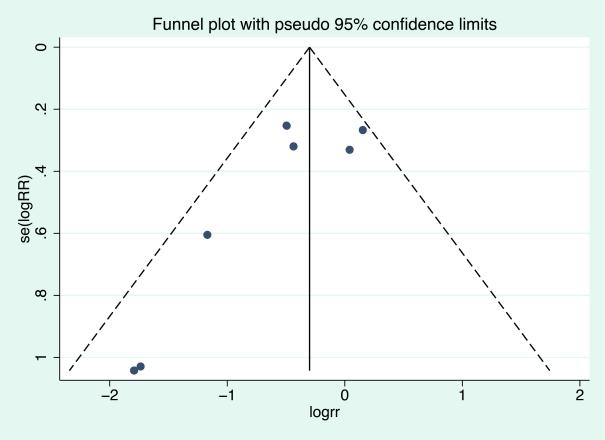 | 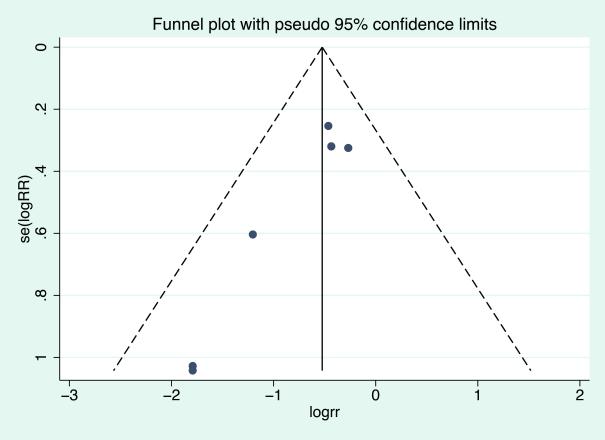 |
| --- | --- |

C


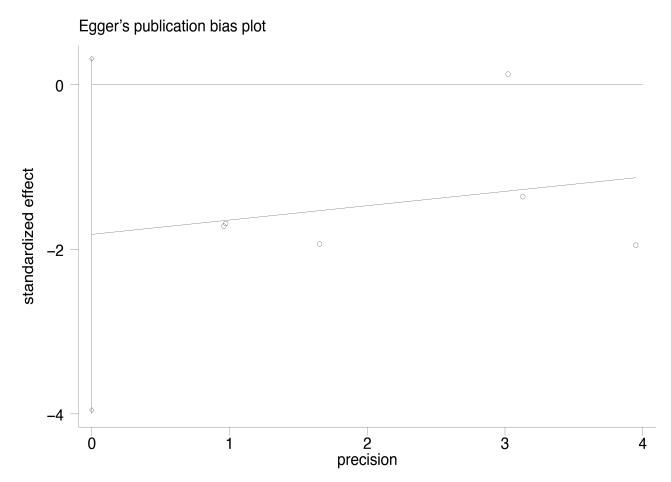


D


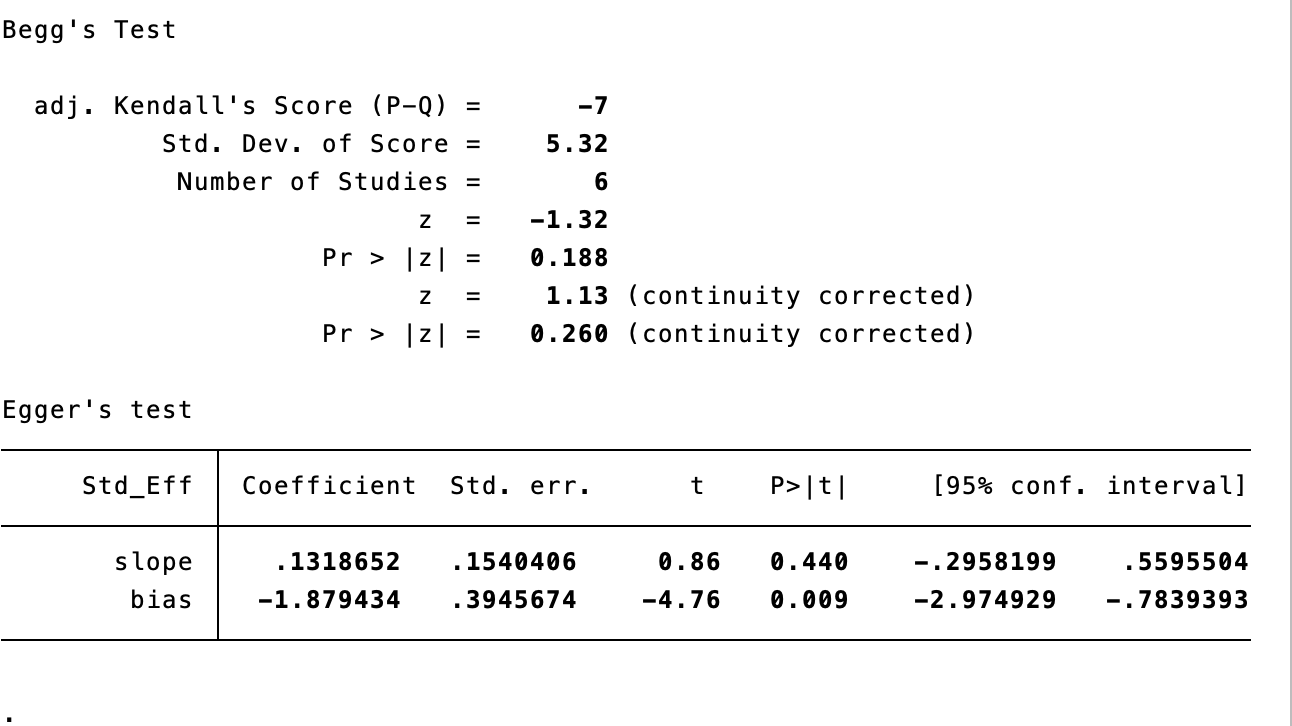


**Figure S3:** Funnel plot(A), Funnel plot after the second sensitivity analysis(B),Egger’s plot(C), Begg’s and Egger’s tests(D) for publication bias for the analysis of the SD.

A B

| 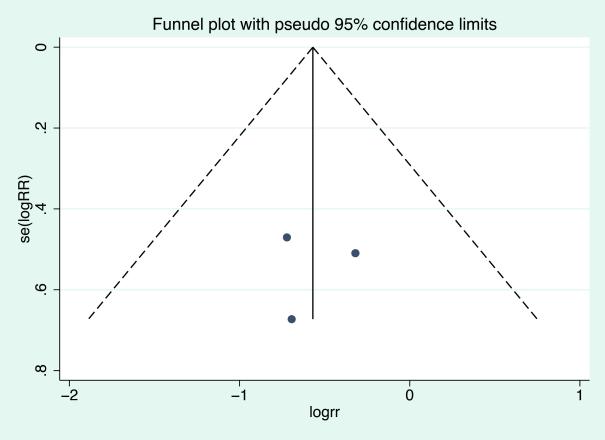 | 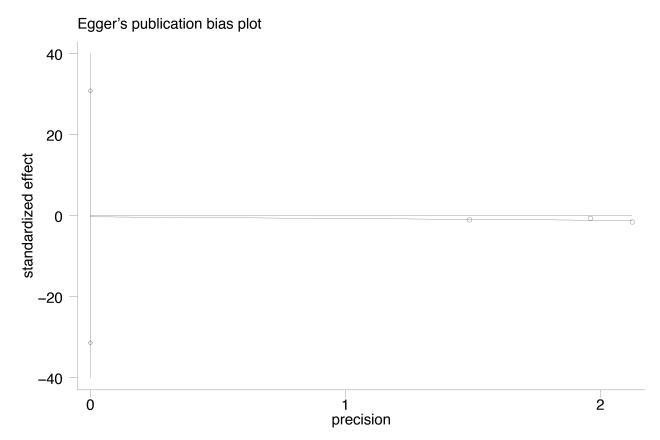 |
| --- | --- |
| C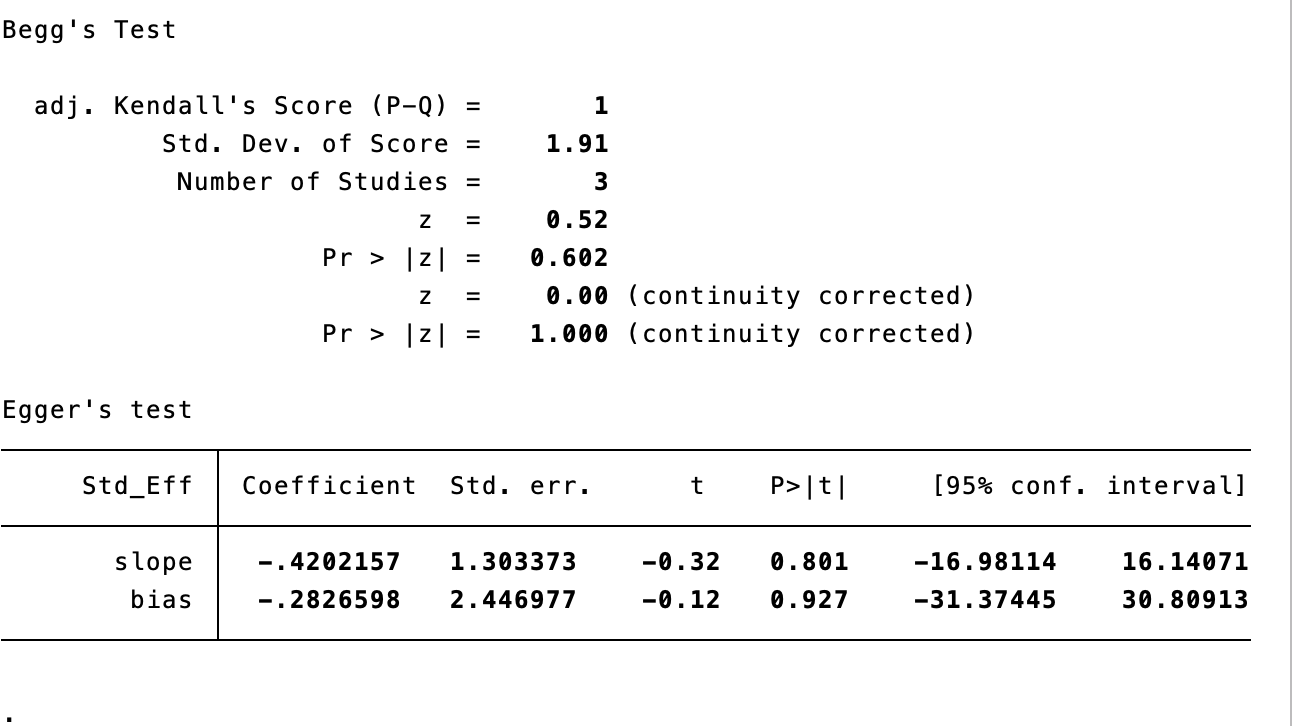 | |

**Figure S4:** Funnel plot(A), Egger’s plot(B), Begg’s and Egger’s tests(C) for publication bias for the analysis of the PD.

A B

| 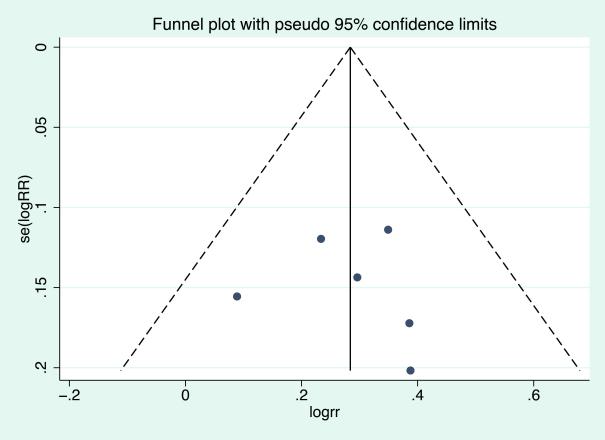 | 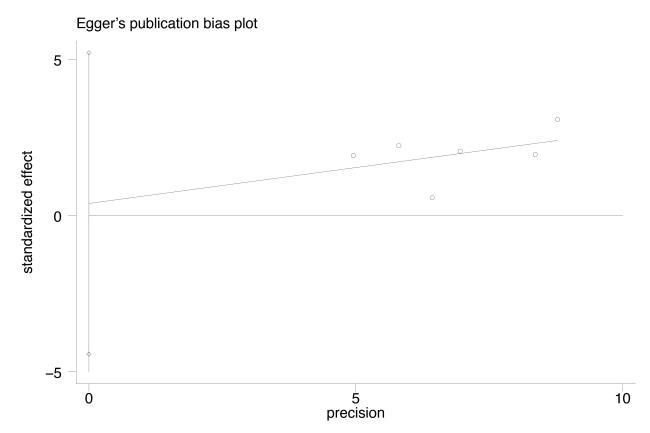 |
| --- | --- |
| C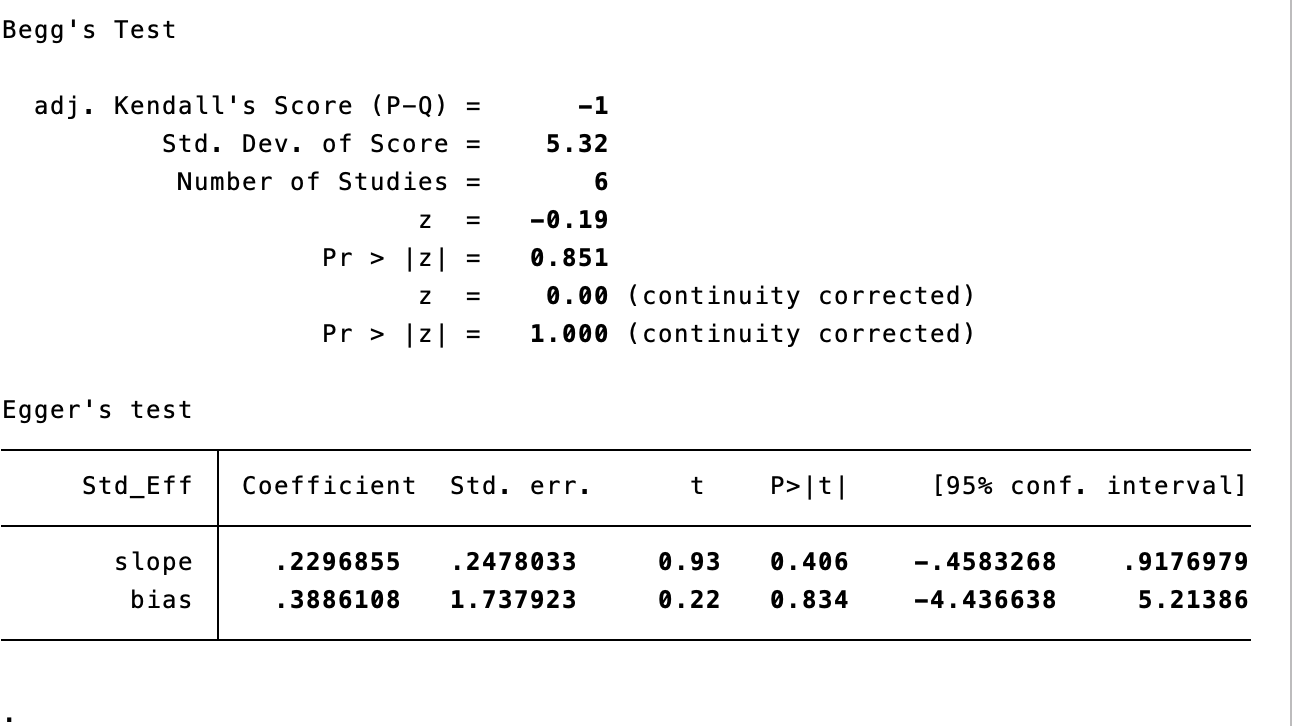 | |

**Figure S5:** Funnel plot(A), Egger’s plot(B), Begg’s and Egger’s tests(C) for publication bias for the analysis of the ORR.

A B

| 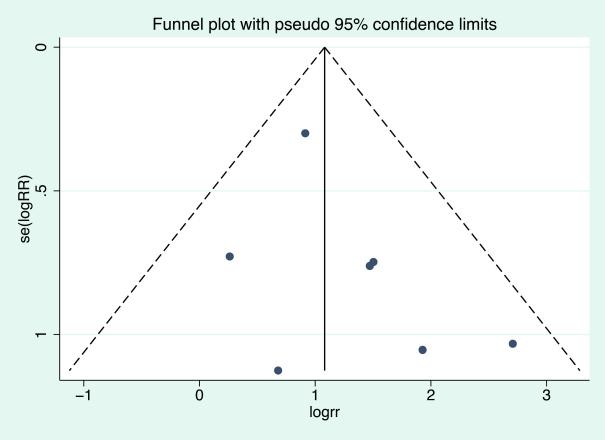 | 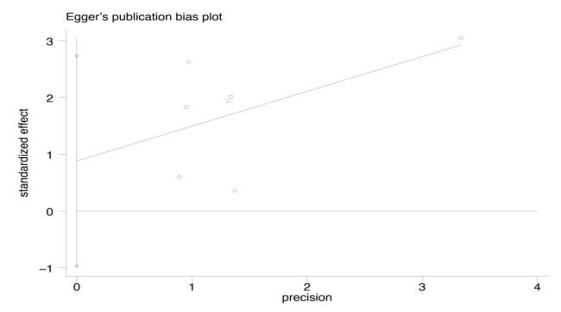 |
| --- | --- |
| C  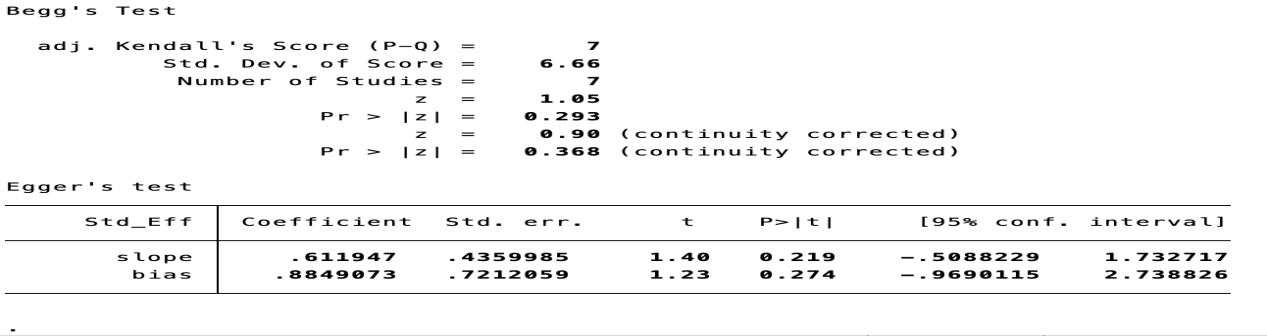 | |

**Figure S6：**Funnel plot(A), Egger’s plot(B), Begg’s and Egger’s tests(C) for publication bias for the analysis of the pCR.

A B

| 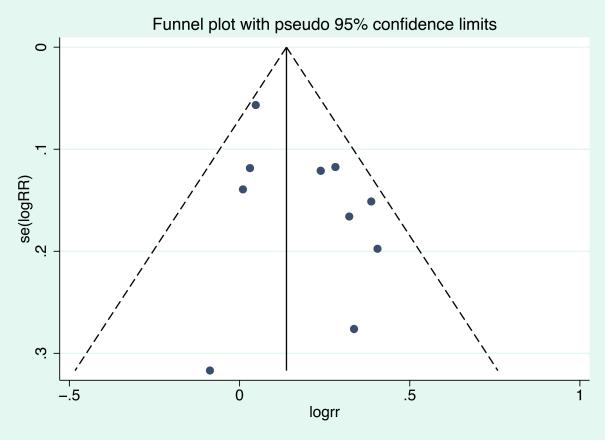 | 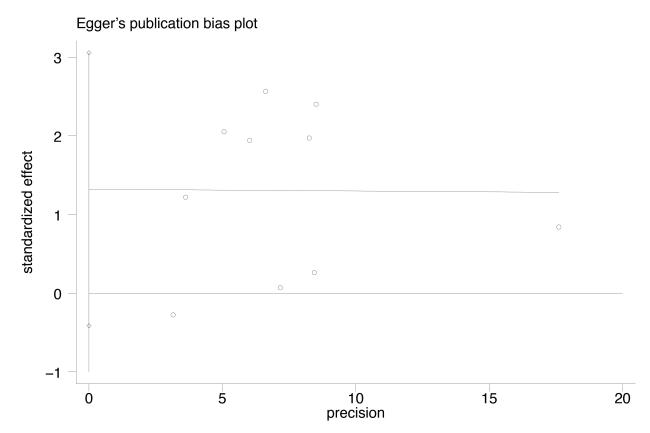 |
| --- | --- |
| C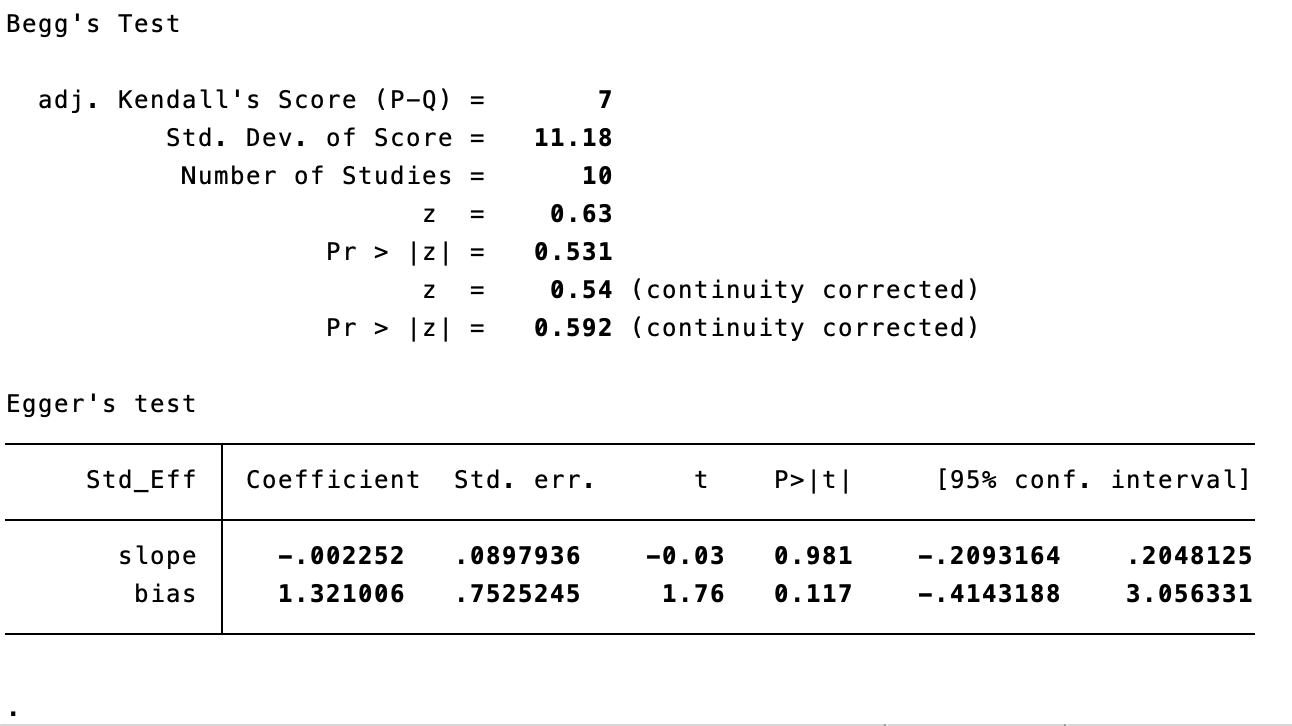 | |

**Figure S7:** Funnel plot(A), Egger’s plot(B), Begg’s and Egger’s tests(C) for publication bias for the analysis of the R0 resection rate.

A B

| **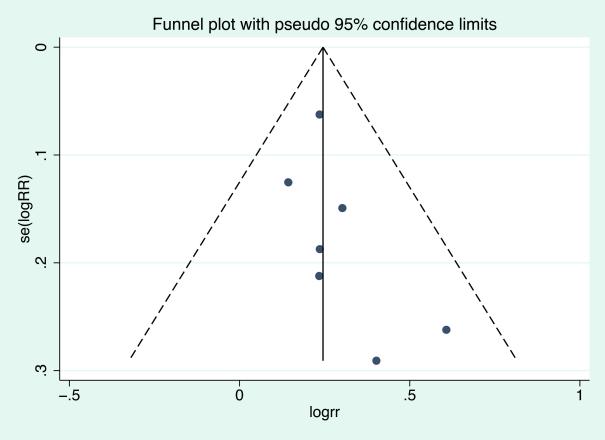** | **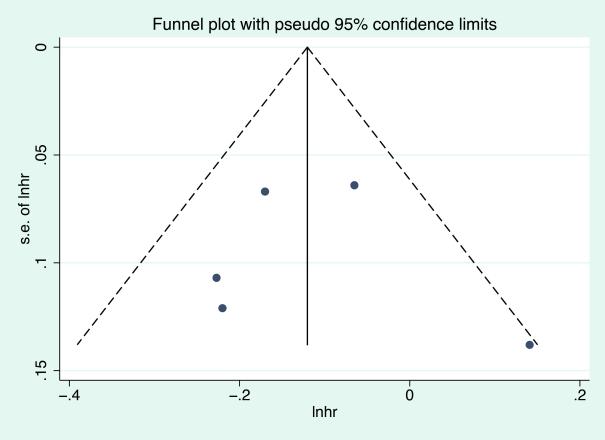** |
| --- | --- |

C

**
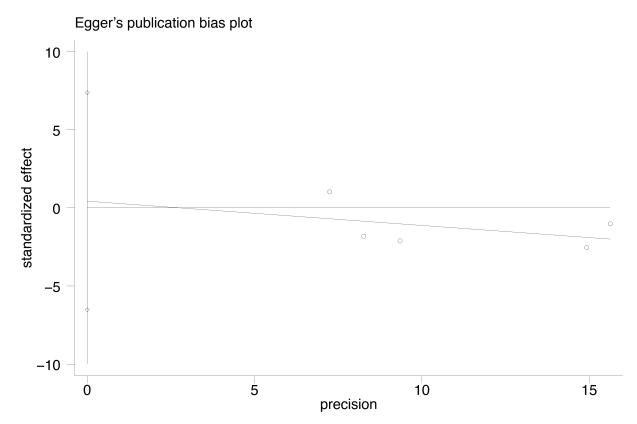
**

D**
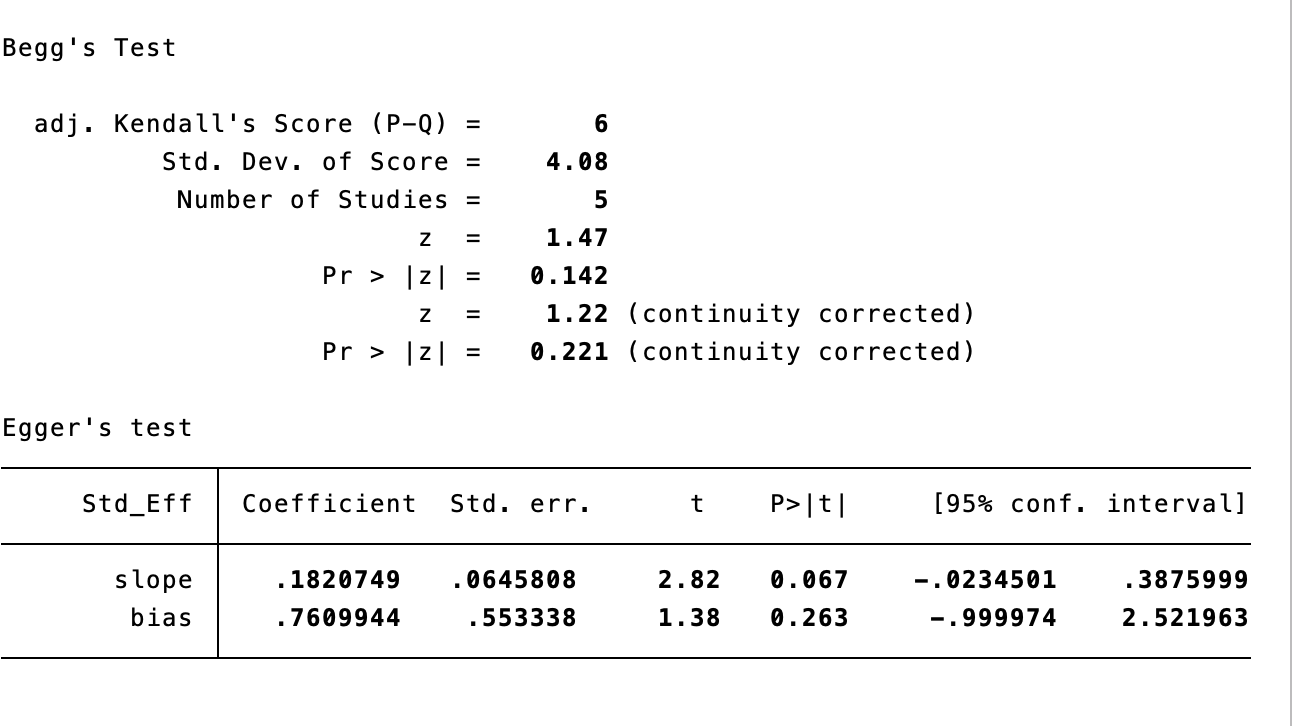
Figure S8：**Funnel plot(A), Funnel plot after the second sensitivity analysis(B),Egger’s plot(C), Begg’s and Egger’s tests(D) for publication bias for the analysis of the 3-year OS.

A B

| 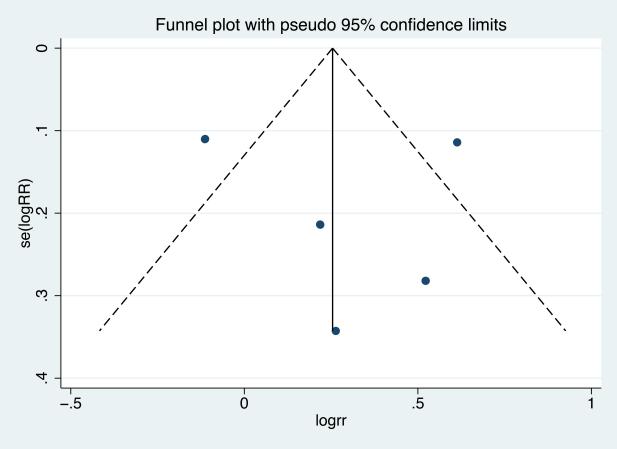 | 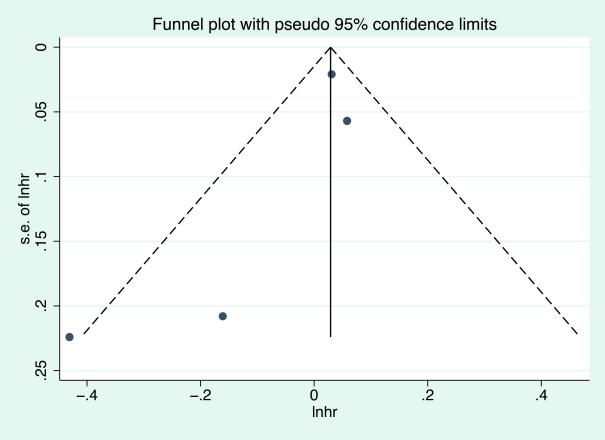 |
| --- | --- |

C


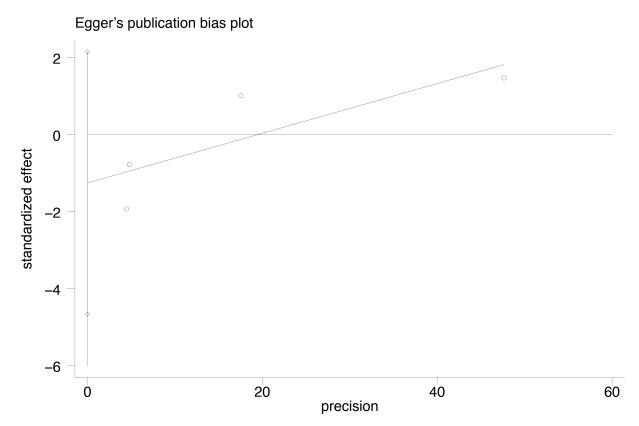


D


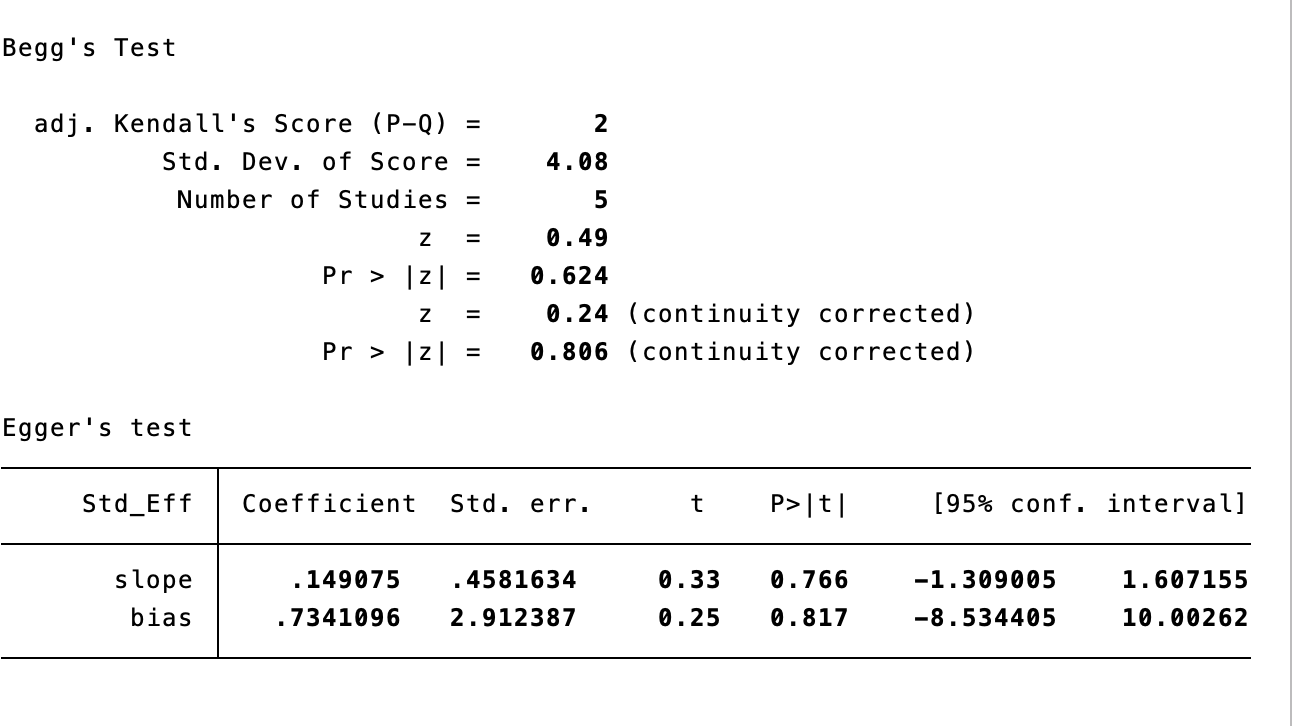


**Figure S9：**Funnel plot(A), Funnel plot after the second sensitivity analysis(B),Egger’s plot(C), Begg’s and Egger’s tests(D) for publication bias for the analysis of the 5-year OS.

A B

| 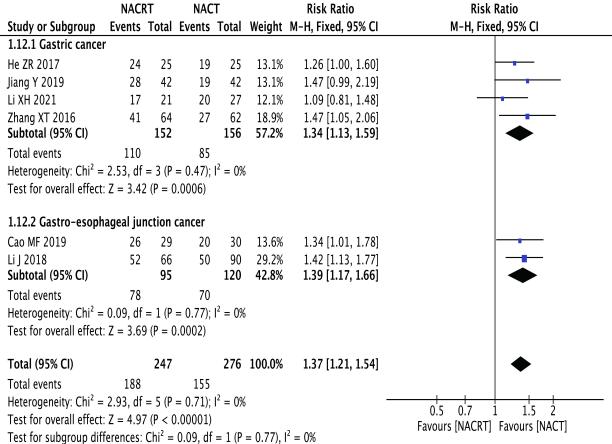 | 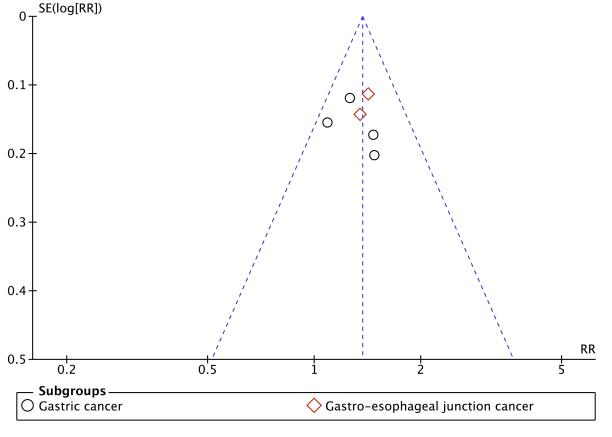 |
| --- | --- |

**Figure S10：**Forest plot(A) and Funnel plot(B) for the subgroup analysis of the ORR.

A B

| **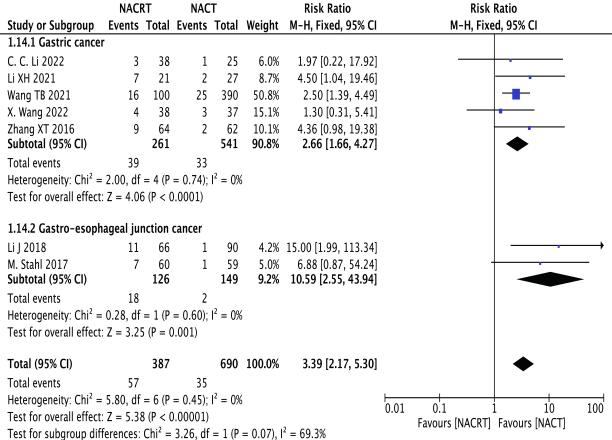** | **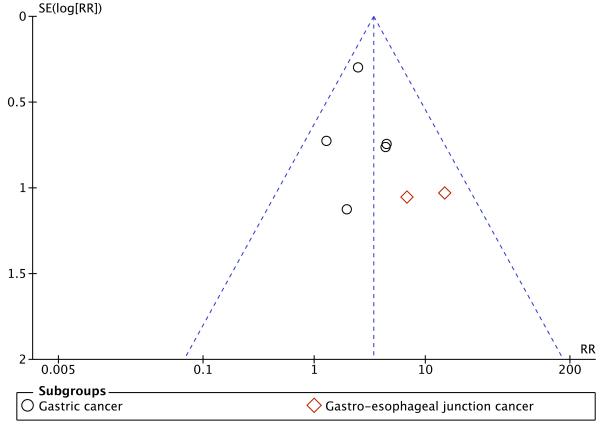** |
| --- | --- |

**Figure S11：**Forest plot(A) and Funnel plot(B) for the subgroup analysis of the pCR.

A B

| 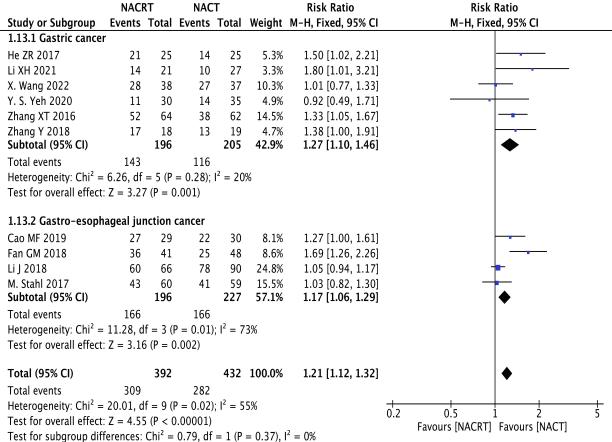 | 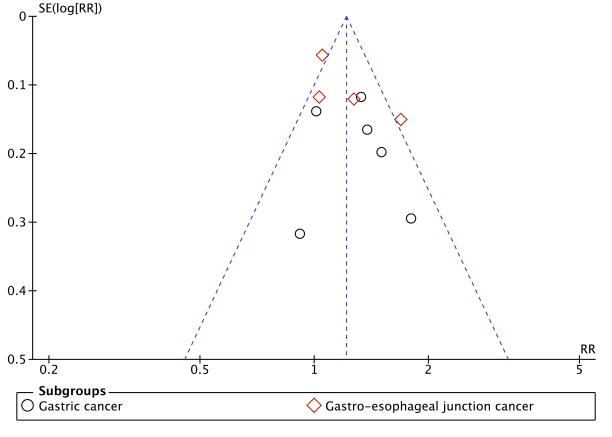 |
| --- | --- |

**Figure S12：**Forest plot(A) and Funnel plot(B) for the subgroup analysis of the R0 resection rate.

A B

| 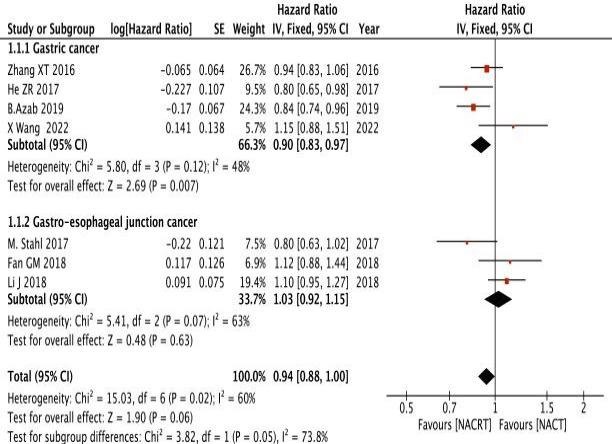 | 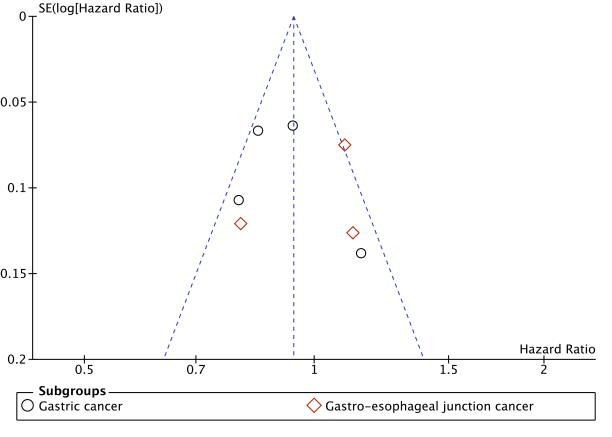 |
| --- | --- |

**Figure S13：**Forest plot(A) and Funnel plot(B) for the subgroup analysis of the 3-year OS.

A B

| 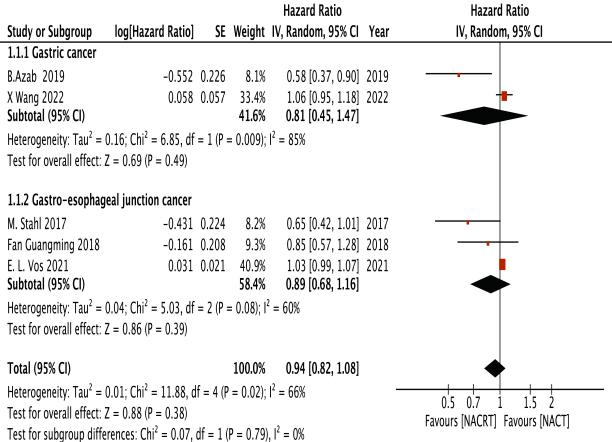 | 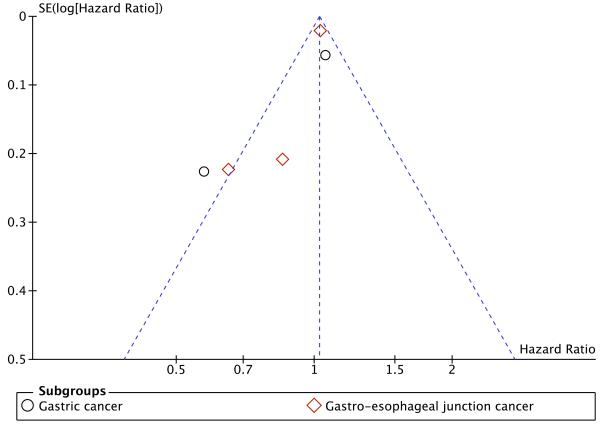 |
| --- | --- |

**Figure S14：**Forest plot(A) and Funnel plot(B) for the subgroup analysis of the 5-year OS.
